# Eukaryotic-Type Ser/Thr Protein Kinase Mediated Phosphorylation of Mycobacterial Phosphodiesterase Affects its Localization to the Cell Wall

**DOI:** 10.3389/fmicb.2016.00123

**Published:** 2016-02-09

**Authors:** Neha Malhotra, Pradip K. Chakraborti

**Affiliations:** CSIR-Institute of Microbial TechnologyChandigarh, India

**Keywords:** signal transduction, cAMP, eukaryotic-type serine/threonine kinase, enzyme activity, mutagenesis, intracellular trafficking, phosphodiesterase

## Abstract

Phosphodiesterase enzymes, involved in cAMP hydrolysis reaction, are present throughout phylogeny and their phosphorylation mediated regulation remains elusive in prokaryotes. In this context, we focused on this enzyme from *Mycobacterium tuberculosis*. The gene encoded by Rv0805 was PCR amplified and expressed as a histidine-tagged protein (mPDE) utilizing *Escherichia coli* based expression system. In kinase assays, upon incubation with mycobacterial Clade I eukaryotic-type Ser/Thr kinases (PknA, PknB, and PknL), Ni-NTA purified mPDE protein exhibited transphosphorylation ability albeit with varying degree. When mPDE was co-expressed one at a time with these kinases in *E. coli*, it was also recognized by an anti-phosphothreonine antibody, which further indicates its phosphorylating ability. Mass spectrometric analysis identified Thr-309 of mPDE as a phosphosite. In concordance with this observation, anti-phosphothreonine antibody marginally recognized mPDE-T309A mutant protein; however, such alteration did not affect the enzymatic activity. Interestingly, mPDE expressed in *Mycobacterium smegmatis* yielded a phosphorylated protein that preferentially localized to cell wall. In contrast, mPDE-T309A, the phosphoablative variant of mPDE, did not show such behavior. On the other hand, phosphomimics of mPDE (T309D or T309E), exhibited similar cell wall anchorage as was observed with the wild-type. Thus, our results provide credence to the fact that eukaryotic-type Ser/Thr kinase mediated phosphorylation of mPDE renders negative charge to the protein, promoting its localization on cell wall. Furthermore, multiple sequence alignment revealed that Thr-309 is conserved among mPDE orthologs of *M. tuberculosis* complex, which presumably emphasizes evolutionary significance of phosphorylation at this residue.

## Introduction

Cells respond to various environmental changes by an orchestra of events and transfer information through the process of signal transduction (McDonough and Rodriguez, 2012). The cascade of events involve identification as well as amplification of signals resulting in gene(s) expression that regulates cellular physiology of an organism and ultimately helps to better acclimatize in the adverse environs for its survival. In this direction, we concentrated on bacterial signaling, especially focussing on pathogens like *Mycobacterium tuberculosis*, the causative agent of the disease tuberculosis (Chevalier et al., 2014). In fact, *M. tuberculosis* is considered to be one of the most successful pathogen as it is capable of withstanding host defense mechanism(s) for its survival (Chevalier et al., 2014; Dey and Bishai, 2014; Orme, 2014). Such compliance might be attributed to its ability to camouflage within the micro-environment of the host and exhibit atypical or unique signal transduction mechanisms. In this context, signaling involving small molecules such as 3′, 5′-cyclic AMP, cyclic di-AMP, cyclic di-GMP etc., are less explored (Hong et al., 2013; Zaveri and Visweswariah, 2013; Manikandan et al., 2014). These cyclic nucleotide levels are controlled by different phosphodiesterases by promoting their hydrolytic degradation and thus, play a crucial role in the process (Shenoy et al., 2005; Hong et al., 2013; Yang et al., 2014).

Analysis of *M. tuberculosis* genome sequence revealed the presence of an exceptionally high 3′, 5′- cAMP synthesizing machinery in the form of adenylate cyclases compared to other micro-organisms. While 16 such genes have been identified in *M. tuberculosis* genome (Shenoy and Visweswariah, 2006; Zaveri and Visweswariah, 2013), there is only one phosphodiesterase (mPDE) enzyme that antagonizes cAMP action through its degradation into 5′AMP (Shenoy et al., 2005; Chakraborti et al., 2011). Apart from cAMP hydrolysis, mPDE is also involved in altering cell wall permeability (Podobnik et al., 2009). The mPDE has already been characterized and its structure was determined using X-ray crystallographic studies (Shenoy et al., 2007). While its N-terminal catalytic domain is responsible for the enzyme activity, the C-terminal region mediates its localization (Podobnik et al., 2009; Matange et al., 2014).

The presence of ortholog of mPDE in the minimal genome of *Mycobacterium leprae* further signifies its importance in mycobacterial signal transduction (Shenoy et al., 2005). PDEs in humans are regulated by phosphorylation through Ser/Thr kinases like cAMP- dependent protein kinase A (PKA) or protein kinase B (PKB), thus influencing their catalytic ability (Macphee et al., 1988; Kitamura et al., 1999; Lindh et al., 2007; Bessay et al., 2008). To our surprise, till date there have been no reports of phosphorylation mediated regulation of any bacterial PDE, including that from *M. tuberculosis*. Although *M. tuberculosis* possesses highly complex as well as dynamic phosphorylation/dephosphorylation machinery comprising of 11 eukaryotic-type Ser/Thr kinases and the cognate phosphatase (Cole et al., 1998; Chakraborti et al., 2011), their involvement in this process is yet unknown. Recent studies indicated that these kinases have several cellular targets and they regulate a plethora of metabolic events in prokaryotes (Pereira et al., 2011). Bioinformatic analysis of these 11 mycobacterial kinases categorized them in five groups, Clades I-V (Narayan et al., 2007). Among these kinases, we concentrated on PknA from *M. tuberculosis* belonging to Clade I. Like other eukaryotic-type Ser/Thr kinases, PknA has a characteristic domain structure and it is predominantly phosphorylated at threonine residues (Chaba et al., 2002; Thakur et al., 2008; Ravala et al., 2015). Available literature also established the role of PknA in morphological changes associated with bacterial cell division (Chaba et al., 2002; Kang et al., 2005) by interacting with FtsZ and MurD. Notably, the FtsZ protein and MurD enzyme play a crucial role in bacterial cytokinesis and peptidoglycan respectively (Thakur and Chakraborti, 2006, 2008).

In this study, we provide evidence that mPDE is transphosphorylated by PknA. Among other mycobacterial Clade I kinases, we noticed PknB and PknL were also able to phosphorylate mPDE albeit with varying magnitude. Utilizing PknA as a representative of mycobacterial eukaryotic-type Ser/Thr kinases, its co-expression with mPDE in *Escherichia coli* yielded a phosphorylated His-tagged protein. LC-MS/MS identified Thr-309 as the phosphorylable residue in this protein, which is located within a short but de-structured C-terminal domain of mPDE (Podobnik et al., 2009; Matange et al., 2014). The mPDE upon expression in *Mycobacterium smegmatis* was recognized by anti-phosphothreonine antibody. Further, our analysis on phosphoablative and phosphomimetic substitutions of the phospho-threonine residue indicated the role of eukaryotic-type Ser/Thr kinase mediated phosphorylation of mPDE for its cell wall association. Interestingly, Thr-309 is conserved amongst mPDE orthologs of *M. tuberculosis* complex portraying its probable evolutionary significance.

## Materials and methods

### Materials

Restriction enzymes were procured from New England Biolabs (USA). Other fine biochemicals like ATP, Tris, NaCl, maltose, MnCl_2_, MgCl_2_, trypsin and dephosphorylated α-casein were procured from Sigma (USA). Antibodies like anti-phosphothreonine (Cell Signalling Technology, USA) and anti-His (GE Healthcare, USA) were commercially available. Phosphodiesterase assay kit was the product of Enzo®Lifesciences (USA). [γ-^32^P] ATP was obtained from Board of Radiation and Isotope Technology (BRIT, India). Desalted oligonucleotides were custom synthesized from Sigma.

### Cloning, expression, and site directed mutagenesis

Different genes were isolated through PCR amplification using genomic DNA from *M. tuberculosis* strain H37Ra as the template. In fact, sequences of mPDE (Rv0805), *pknB* (Rv0014c), and *pknL* (Rv2176) were identical at nucleotide level between pathogenic (H37Rv) and non-pathogenic (H37Ra) strains of *M. tuberculosis*. For amplification of mPDE gene, PCR (denaturation: 5 min at 95°C; reaction: 1 min at 95°C, 0.5 min at 58°C, 0.5 min at 72°C for 29 cycles; final extension: 10 min at 72°C) was carried out using Herculase DNA polymerase enzyme, primers incorporating restriction sites (CN15: 5′- ACG TACGAATTCCATATGCATAGACTTAGGG-3′ and CN16: 5′- CTAGACAAGCTTTCAGTCGACGGGACTTC-3′) and template DNA. Amplified DNA fragments were cloned either in pET-28c/pVV2 (at NdeI/HindIII sites for mPDE) or in pMAL-c2X (BamHI/HindIII for PknB or PknL) and maintained as pET-mPDE/pVV2-mPDE/pMAL-PknB/pMAL-PknL in *E. coli* strain DH5α. PknB (PCR amplified using 5′- AATTAGGATCCCAT ATGACCACCCCTCCC-3′ and 5′- ACTGCAAGCTTCTA CTGGCCGAACCT-3′ primers) and PknL (PCR amplified using 5′- AACTTGGATCCCATATGGTCGAAGCTGGCACG-3′ and 5′- GGAATTAAGCTTTTACAGCAGGCCGCTCAGGTTG-3′ primers) constructs were available in the lab. The pET-mPDE construct was transformed in *E. coli* strain BL21(DE3) for protein expression and purification. Besides these, pMAL-PknA/-PknA-core/-PknA-K42N, p19kpro-PknA/-PknA-K42N (Rv0015c or its variant containing amino terminal 338 residues or its PknA-K42N mutant either in pMAL or p19kpro) and pMAL-PPP (Rv0018c in pMAL) used in this study were described earlier (Chaba et al., 2002; Thakur and Chakraborti, 2006, 2008; Thakur et al., 2008; Ravala et al., 2015).

mPDE-T309A, mPDE-T309D and mPDE-T309E mutants were generated by employing overlap extension PCR method (Ho et al., 1989) using pET-mPDE DNA as the template. Primary PCR was performed using two sets of primers for each mutant (set 1: terminal forward, CN15 and internal reverse, CN54: 5′-TGCCGAGGACGCCAGCACCAT-3′; set 2: internal forward, CN53: 5′-ATGG TGCTGGCGTCCTCGGCA-3′ and terminal reverse, CN16 for mPDE-T309A; set1: CN15, and CN57: 5′- ATGGTGCTGGACTCCTCGGCA-3′; set2: CN58: 5′- TGCCGAGGAGTCCAGCACCAT-3′ and CN16 for mPDE-T309D; set 1: CN15 and CN59: 5′- ATGGTGCTGGAATCCTCGGCA-3′; set 2: CN60: 5′- TGCCGAGGATTCCAGCACCAT-3′ and CN16 for mPDE-T309E). For secondary PCR, two independent fragments generated in this way were mixed at equi-molar concentrations (1:1), amplified using primers (CN15 and CN16) and cloned in pET-28c or pVV2 vectors.

Recombinant proteins expressed in *E. coli* were purified following protocol described earlier with slight modifications (Chaba et al., 2002). Briefly, fresh culture (1% inoculum from overnight cultures) using LB broth with appropriate antibiotics was grown till OD_600_ of 0.6–0.8, induced with 0.4 mM IPTG and further incubated at 37°C for 3 h. This was followed by harvesting and resupension of cell pellet in lysis buffer (50 mM Tris-HCl, pH 7.5 and 150 mM NaCl) along with protease inhibitors (1 mM phenylmethylsulfonylfluoride, 1 μg/ml pepstatin and 1 μg/ml leupeptin). Cells were lysed by sonication (over ice; 10 s “on” and 10 s “off” cycles for 10 min; 20% amplitude) and centrifuged at 18,000 × g at 4°C for 60 min. The supernatant fraction was loaded onto Ni-NTA affinity column equilibrated with 10 mM imidazole in lysis buffer. Column was washed with 20 mM imidazole in lysis buffer and protein was eluted using 50–200 mM imidazole in lysis buffer.

The mPDE or mPDE-T309A/D/E variants were also expressed in *M. smegmatis* strain mc^2^155 following its cloning in pVV2 vector (Dhiman et al., 2004; Thakur and Chakraborti, 2008) at NdeI/HindIII sites (pVV2-mPDE or pVV2-mPDE-T309A/D/E). These constructs were transformed by electroporation in *M. smegmatis* strain mc^2^155 following standard procedure (Thakur and Chakraborti, 2008). Cells harboring wild-type or mutants in pVV2 vector constructs were inoculated in Middlebrook 7H9 media enriched with OADC (10%) and Tween-80 in presence of kanamycin (25 μg/ml) and grown at 37°C at 200 rpm for 24 h (OD_600_ of ~0.8). Following their inoculation (1% inoculum) in LB media supplemented with kanamycin (25 μg/ml) at 37°C till OD_600_ of 0.6-0.8, cells were induced with H_2_O_2_ (0.002%) and grown further in same conditions for 6 h. For purification of recombinant proteins, cells were then processed as mentioned above for *E. coli* expression system except for slightly altered procedure for sonication (20 s “on,” 40 s “off” 40% amplitude for 60 min over ice). Finally, protein was eluted with 250 mM imidazole (in lysis buffer), aliquoted in small fractions and stored at −80°C till further use. For preparation of sub-cellular fractions, cellular lysis was accomplished by 2-3 passages through French Press at 1500 psi followed by sonication (all parameters same as mentioned above except time for 30 min). The lysate was then centrifuged at 8000 × g at 4°C for 5 min for removal of unbroken cells/cellular debris. Centrifugation was repeated at 18,000 × g at 4°C for 1 h to separate pellet from supernatant. The obtained pellet after suspending in lysis buffer was further extracted with 2% SDS (65°C for 2 h) and the supernatant fraction obtained after centrifugation (18,000 × g at 25°C for 45 min) was used as the cell wall fraction (Wang et al., 2000; Dave et al., 2002; Gibbons et al., 2007). On the other hand, supernatant obtained after cell lysis was ultracentrifuged at 110,000 x g at 4°C for 2 h. Orange-brown pellet obtained was used as the membrane fraction after resuspending in lysis buffer (400 μl), while the supernatant was the cytosolic part.

### Kinase assay

Transphosphorylation of His_6_-mPDE by PknA (full length construct containing 431 amino acids or N-terminal 338 amino acid containing catalytic core; Thakur et al., 2008) or kinase dead variant of PknA (PknA-K42N) or PKnB or PknL was assessed utilizing an *in vitro* kinase assay reported earlier (Chaba et al., 2002; Lakshminarayan et al., 2008). Briefly, PknA (0.28 μM for full length or 0.5 μM for catalytic core) or PknB (0.23 μM) or PknL (0.57 μM) or PknA-K42N (0.44 μM) in 1X kinase buffer (50 mM Tris-Cl, pH 7.5 or 6.8 for PknL/50 mM NaCl containing MnCl_2_, 10 mM for PknA/PknL/PknA-K42N or 3 mM for PknB, and 10 mM MgCl_2_ only for PknL) and 2 μCi of [γ-^32^P] ATP was incubated in the presence of mPDE (10 μg or 13.2 μM/reaction) at 25°C (30°C for PKnL) for 30 min (total reaction volume = 20 μl). To monitor PPP mediated de-phosphorylation, it was added to the reaction mix (10 μg or 5 μM/reaction) at this step and further incubated for 75 min at 37°C. The reaction was stopped by adding 5X SDS buffer. Samples were resolved in 10% SDS-PAGE; gels were stained with Coomassie Brilliant Blue. Gels were analyzed in a phosphoimaging device (Fuji Film model FLA 9000) and/or exposed to Kodak X-Omat/AR film for autoradiography.

### Western blotting

Purified proteins (usually 1 μg) or whole cell lysates (usually 25 μg) were resolved on 10% SDS-PAGE, transferred (120 V) to nitrocellulose membrane (0.45 μ) using mini-transblot apparatus (Bio-Rad, USA), probed with either anti-His (1:3000) or anti-RpoB (1:3000) or anti-phosphothreonine (1:1000) or anti-PknA (1:1000) or anti-mPDE (1:1000) and anti-mouse (for anti-His and anti-RpoB antibodies) or anti-rabbit IgG (1:5000) as primary and secondary antibodies, respectively. For monitoring *in vivo* phosphorylation, pET-mPDE or pET-mPDE-T309A was co-transformed with pMAL-PknA or p19kpro-PknA or pMAL-PknB or pMAL-PknL in *E. coli* BL21(DE3) cells and their expression as a phosphorylated protein was detected employing western blotting using an anti-phosphothreonine antibody as described earlier (Thakur and Chakraborti, 2006). Proteins were finally detected with luminata substrate (Millipore, USA) and developed on X-ray films (Kodak, USA) following manufacturer's recommended protocol.

### Phosphodiesterase enzyme activity

PDE assays were performed using an assay kit of Enzo®Lifesciences, which employs colorimetric detection of hydrolysis of standard 3′, 5′- cAMP (substrate) by cyclic nucleotide phosphodiesterase. Briefly, the catalytic activity of mPDE (3 μg or 3.96 μM) mediated hydrolysis of 3′, 5′- cAMP (0.05 – 0.8 mM) into 5′AMP was monitored in presence of Mn^2+^ (200 μM) at 30°C in a coupled assay following its conversion into adenosine and phosphate catalyzed by 5′ nucleotidase enzyme (0.5 kU/μl). Phosphate such generated was detected by BIOMOL GREEN™ dye at OD of 620 nm at room temperature. Standard curve was prepared by using 5′AMP (0.25 to 3 nmol) in PDE assay buffer that catalyzes its cleavage by 5′ nucleotidase enzyme.

### Mass spectrometry

Samples for mass spectrometry were prepared following the manufacturer's recommended protocol using proteomics grade dimethylated trypsin from porcine pancreas (Sigma, USA). Briefly, Ni-NTA purified proteins in 100 mM ammonium bicarbonate buffer, pH 8.4 was digested in-solution with trypsin at 37°C for 16 h. For cell wall fractions, samples after resolving in SDS-PAGE were eluted in 200 mM ammonium bicarbonate buffer, pH 8.4 containing 40% acetonitrile and then trypsinized (37°C for 16 h) in 40 mM ammonium bicarbonate buffer, pH 8.4 containing 9% acetonitrile. This was followed by running samples on UHPLC system (1290 series, Agilent Technologies) with C8 column coupled to 6550 iFunnel QTOF LC/MS (Agilent Technologies, USA). Electron spray ionization (ESI) was operated in positive ion mode with an MS and MS/MS acquisitions rate of 2 and 4 spectra/second respectively. Peptide sequences were identified utilizing ProteinPilot™ software (http://www.absciex.com/products/software/proteinpilot-software) and Mascot server (http://www.matrixscience.com) as well.

### Sequence analysis and phylogeny

Reciprocal best blast hits were procured from MycoRRdb database (Midha et al., 2012), multiple sequence alignment was done by Muscle program (Edgar, 2004) and phylogenetic tree was constructed by neighbor joining method (Bootstrap value of 500) using Mega 6.0 software (Tamura et al., 2013).

## Results

### Phylogenic placement of mPDE

**Figure 1:**
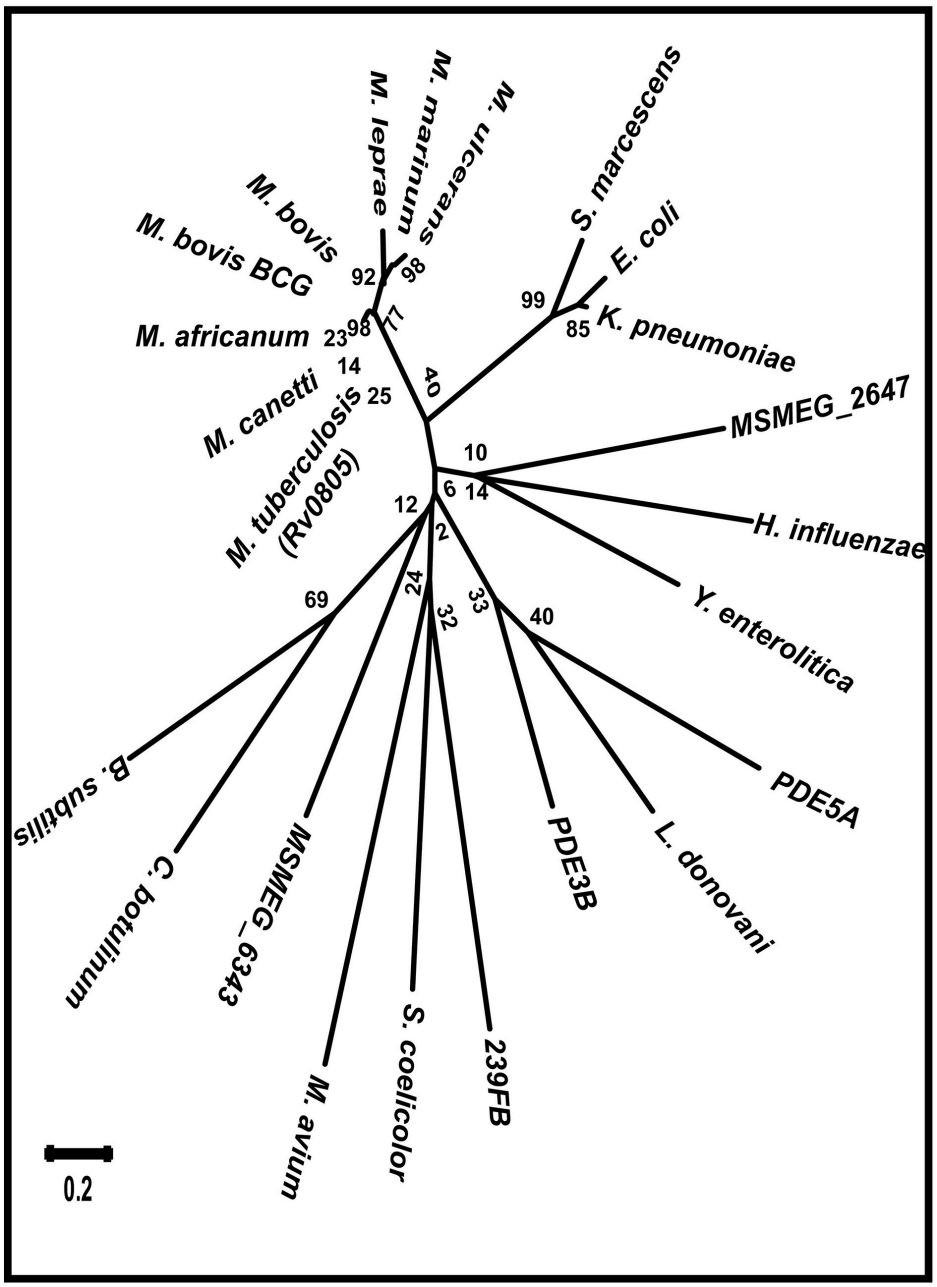
**Mycobacterial PDEs are phylogenetically distinct**. PDE sequences from organisms of different taxa were used to obtain phylogenetic relationship of mPDE. PDE sequences from few mycobacterial species, Gram positive and negative bacteria and eukaryotes were used to obtain phylogenetic tree of radiation type by neighbor joining method using Mega 6.0 software. Numerical values on the nodes represent the bootstrap confidence and scale denotes evolutionary distance.

We initiated our studies to have phylogenetic insights on the placement of mPDE in relation to other bacteria as well as eukaryotes. For this, the gene encoding mPDE (Rv0805) was used as a query to search for the best blast hits in different mycobacterial species. In order to have a wider taxonomic distribution, we extended search in NCBI and Pubmed databases to obtain sequences for few phosphodiesterases present in eukaryotes, gram positive and gram negative bacteria. Multiple sequence alignment using Muscle algorithm followed by phylogenetic tree construction using neighbor-joining method was performed for all the sequences by Mega 6.0 software. Radiation tree shows that mPDE has a unique and distinct taxonomic distribution (Figure 1). Interestingly, orthologs of mPDE are present only in members of *M. tuberculosis* complex (viz. *M. tuberculosis, Mycobacterium africanum, Mycobacterium bovis, Mycobacterium canettii, M. leprae, Mycobacterium ulcerans)* along with other slow growing bacilli (*Mycobacterium marinum)*. It is pertinent to mention that phosphodiesterases in *Mycobacterium avium* and *M. smegmatis* (MSMEG_2647 and MSMEG_6343), are distantly placed from *M. tuberculosis* (Figure 1).

### Phosphorylation of mPDE by mycobacterial eukaryotic-type Ser/Thr kinases

**Figure 2:**
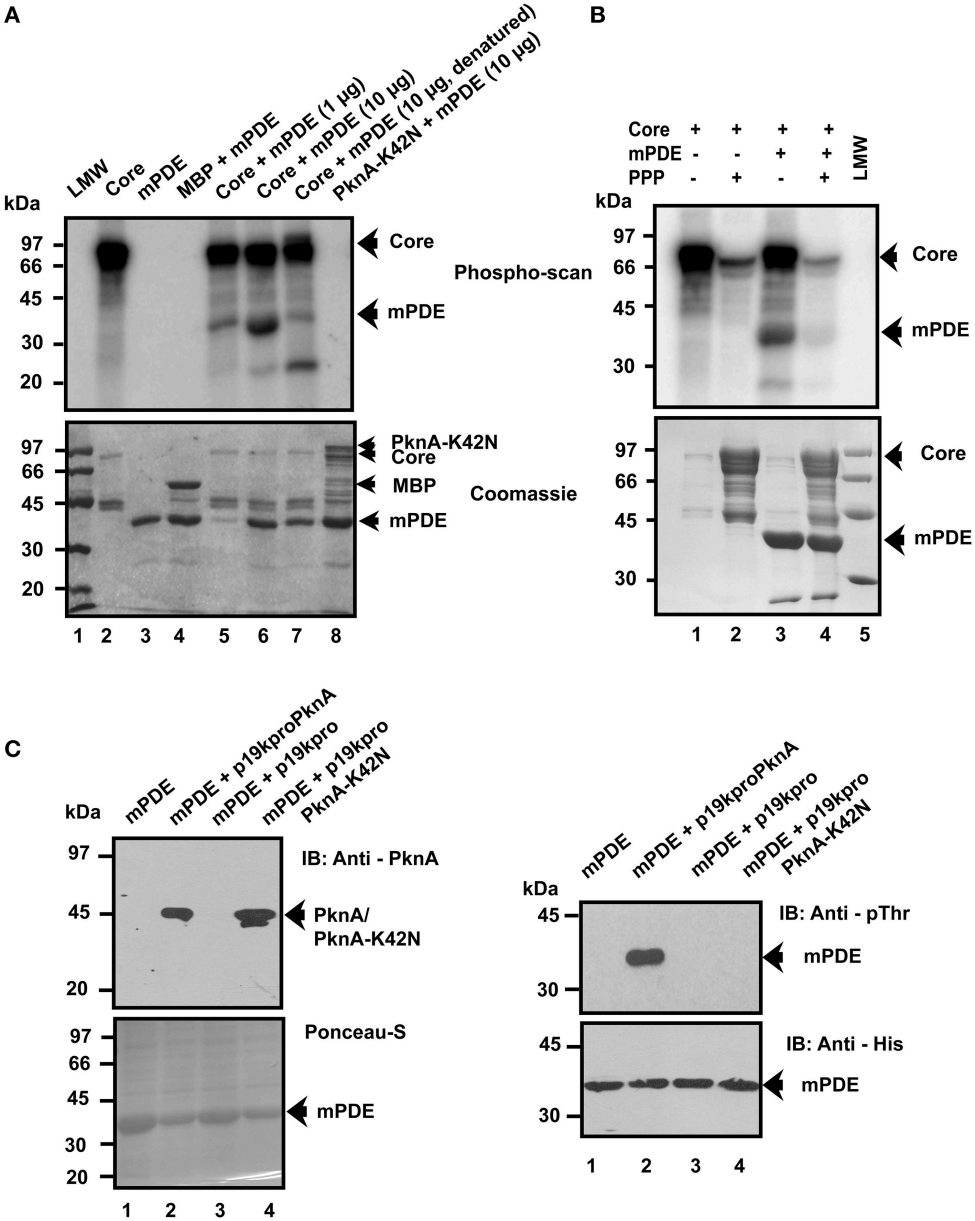
**Phosphorylation of mPDE by PknA. (A)** *In vitro* phosphorylation of mPDE. MBP (0.89 μM)/MBP-PknA Core (0.5 μM)/MBP-PknA-K42N (0.44 μM) were briefly incubated with Ni-NTA purified 1 (1.32 μM) or 10 μg (13.2 μM) mPDE. Samples were processed as described under “Materials and Methods.” Coomassie stained radioactive gel provided in lower panel was used as a loading control. **(B)** *In vitro* dephosphorylation of mPDE. Phosphorylated proteins were dephosphorylated following incubation with PPP (10 μg or 5 μM) at 37°C for 75 min. Coomassie stained radioactive gel served as the loading control (lower panel). **(C)** *In vivo* phosphorylation of mPDE. BL21(DE3) cell lysates expressing His_6_-mPDE along with either p19kpro-PknA or empty vector (p19kpro) or kinase dead mutant (p19kpro-PknA-K42N) were probed with anti-PknA antibody (left upper panel) while ponceau-S stained blot (left lower panel) served as a loading control. Anti-PknA antibody used in this study was generated in our laboratory (Thakur and Chakraborti, 2008). His_6_-tagged mPDE protein purified from these cells was immunoblotted with anti-phosphothreonine (right upper panel) or anti-His (right lower panel) antibody. Notations used: LMW, low molecular weight marker; PPP, phospho serine/threonine phosphatase.

A few of the eukaryotic phosphodiesterases (PDE3b, PDE4, PDE5a, and PDE10A etc.) are functionally regulated by PKA/PKB induced phosphorylation (Macphee et al., 1988; Kitamura et al., 1999; Liu and Maurice, 1999; Kotera et al., 2004; Lindh et al., 2007; Bessay et al., 2008; Charych et al., 2010). However, no such phosphorylation mediated regulation of PDE enzyme has ever been reported in any prokaryote. To evaluate this aspect, mPDE protein was purified to substantial homogeneity using Ni-NTA affinity based column chromatography, which on resolving in 10% SDS-PAGE and staining, yielded a major band with molecular mass of 38 ± 6 (Mean ± SD, *n* = 3) along with slight contamination as discussed previously (Shenoy et al., 2005). We initiated our studies using PknA as a representative of eukaryotic-type Ser/Thr kinases in *M. tuberculosis* to study phosphorylation of mPDE. Accordingly, *in vitro* kinase assay was performed wherein mPDE was incubated in presence and absence of MBP-tagged PknA core [amino terminal 338 residues containing catalytic and juxtamembrane domains of the kinase that exhibited enzymatic activity (Thakur et al., 2008)]. As shown in Figure 2A, PknA core (0.5 μM) was capable to trans-phosphorylate mPDE with an increase in phospho-signal between 1 (1.32 μM) and 10 μg (13.2 μM) of protein (lanes 5 and 6). However, phospho-signal was lost significantly due to deficit kinase-substrate interactions when an inactive mPDE (denatured by boiling at 95°C for 5 min) was used in the assay (lane 7). Similarly, no signal was obtained when it was incubated with kinase dead mutant MBP-PknA-K42N (lane 8). Furthermore, incubation of phosphorylated protein (obtained after kinase assay) with only Ser/Thr phosphatase PPP, at 37°C for 75 min led to loss of phospho-signal of mPDE (Figure 2B; compare lanes 3 and 4). Notably, Coomassie stained radioactive gels were used as loading controls for the experiments (Figures 2A,B, lower panels).

To reveal the existence of phosphorylation of substrate protein (mPDE) *in vivo*, mPDE was co-expressed with full length PknA or kinase-dead variant PknA-K42N in *E. coli* BL21(DE3) cells utilizing two incompatible vector systems (pET-28c and constitutive expression vector p19kpro bearing same origin of replication). Expression of PknA or PknA-K42N (along with mPDE) was confirmed in *E. coli* cell lysates (5 μg protein/lane) by western blotting using anti-PknA antibody (observed molecular mass = ~45 kDa, lanes 2 and 4, Figure 2C upper left panel and also see Figure S1). Ponceau-S staining of same blot served as a loading control where mPDE bands in all lanes were stained (Figure 2C, lower left panel and also see Figure S1). Despite loading same amount of total protein in each lane (5 μg), variation in expression levels of mPDE was observed in Ponceau-S stained blot, which was very likely the result of differential selection pressure of two different antibiotics (kanamycin for lane 1 while kanamycin plus hygromycin for lanes 2, 3, and 4 of Figure 2C) and consistent with the previous reports (Yang et al., 2001; Thakur and Chakraborti, 2006; Thakur et al., 2008; Dar and Chakraborti, 2010). Moreover, since the translation machinery of the cell was also engaged in simultaneous expression of two different proteins (mPDE and PknA/PknA-K42N; Figure 2C, lanes 2 and 4), their levels of expression were hard to control. Ni-NTA purification of lysates, followed by immunoblotting revealed that co-expression of mPDE with PknA, but not with the kinase-dead variant PknA-K42N, was a phospho-protein recognized by anti-phosphothreonine antibody (Figure 2C, upper right panel lane 2). The presence of mPDE was also confirmed in western blotting of samples probed with anti-His antibody (Figure 2C, lower right panel).

**Figure 3:**
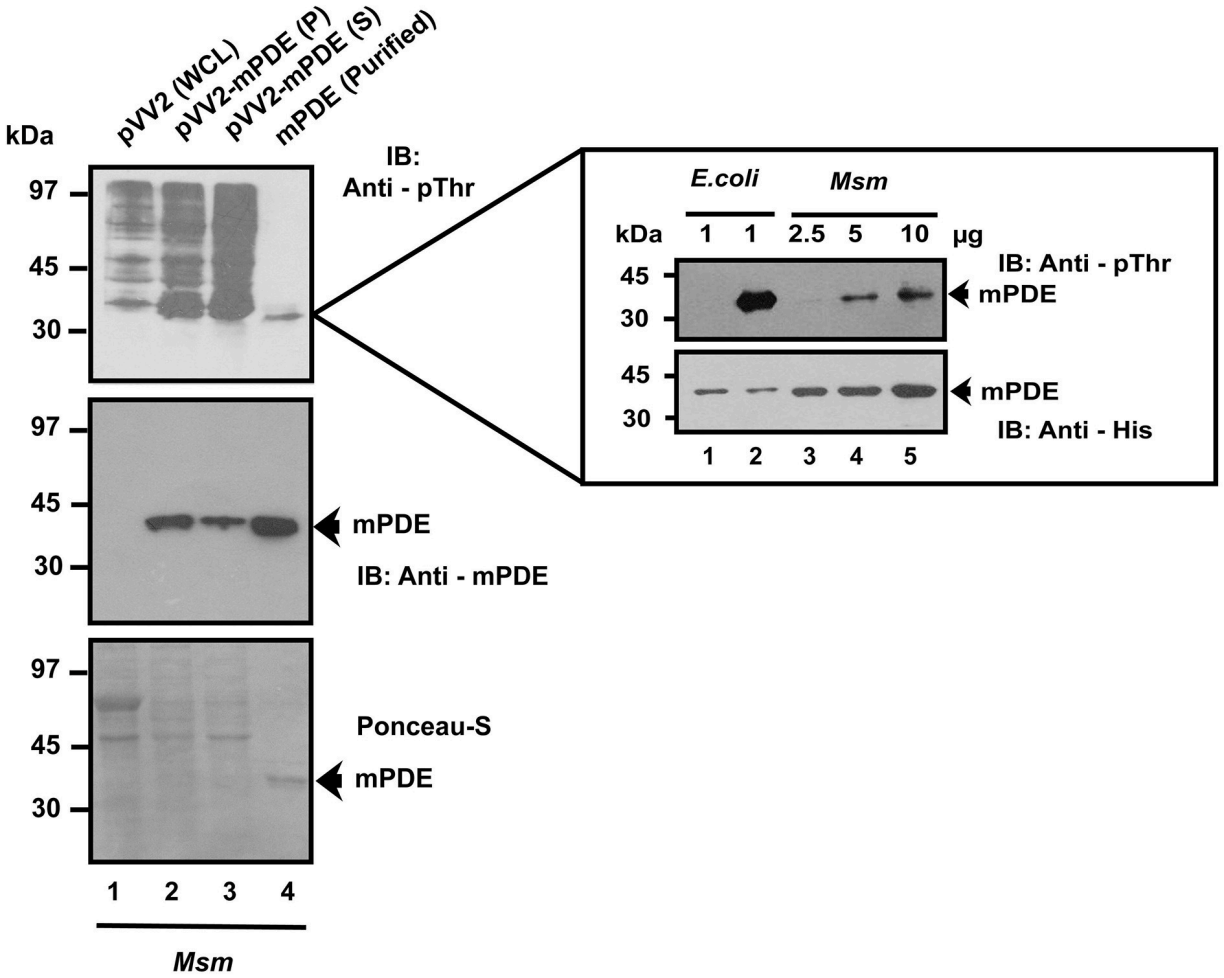
**Phosphorylation of mPDE by mycobacterial endogenous kinases**. His_6_-mPDE cloned in pVV2 vector was expressed and purified from *M. smegmatis*. Expression and phosphorylation status of mPDE in different cellular fractions (P: pellet and S: supernatant) or Ni-NTA purified form (mPDE) was determined by immunoblotting with anti-mPDE (middle panel) and anti-phosphothreonine antibody (top panel). Whole cell lysate of *M. smegmatis* was used a negative control for mPDE expression (lane 1). Polyclonal antibody against mPDE was raised in rabbit following the method described elsewhere (Sarin et al., 2001). Ponceau-S stained blot served as a loading control for the same. Inset shows dose dependent increase in phospho-signal of mPDE isolated from *M. smegmatis* (lanes 3, 4, and 5). Western blotting with anti-His antibody unlike the other case (with anti-mPDE antibody) was done to prevent saturation of blot at higher amounts of mPDE. Unphosphorylated and phosphorylated mPDE protein purified from *E. coli* strain BL21(DE3) were used as internal controls (inset, lanes 1 and 2). Notations used: WCL, whole cell lysate; *Msm, M. smegmatis*.

We further expressed mPDE as His-tagged protein in *M. smegmatis*, which has endogenous sensor kinases but no orthologs of mPDE (Etienne et al., 2005; Narayan et al., 2007; Podobnik et al., 2009). In fact, *M. smegmatis* is often used as a model in carrying out genetic studies with *M. tuberculosis* (Thakur and Chakraborti, 2008; Baronian et al., 2015; Zhou et al., 2015). Western blotting using anti-phosphothreonine antibody recognized His-tagged mPDE protein that phosphorylated in cellular extracts (10 μg/lane, Figure 3, lanes 2 and 3) and in purified form (2.5 μg, Figure 3, lane 4). Authenticity of mPDE protein was confirmed utilizing polyclonal anti-mPDE antibody (Figure 3, middle panel). The Ni-NTA purified mPDE protein exhibited a dose-dependent increase in band intensity (inset, Figure 3). Thus, our results strongly indicated mPDE as a substrate of eukaryotic-type Ser/Thr protein kinases.

**Figure 4:**
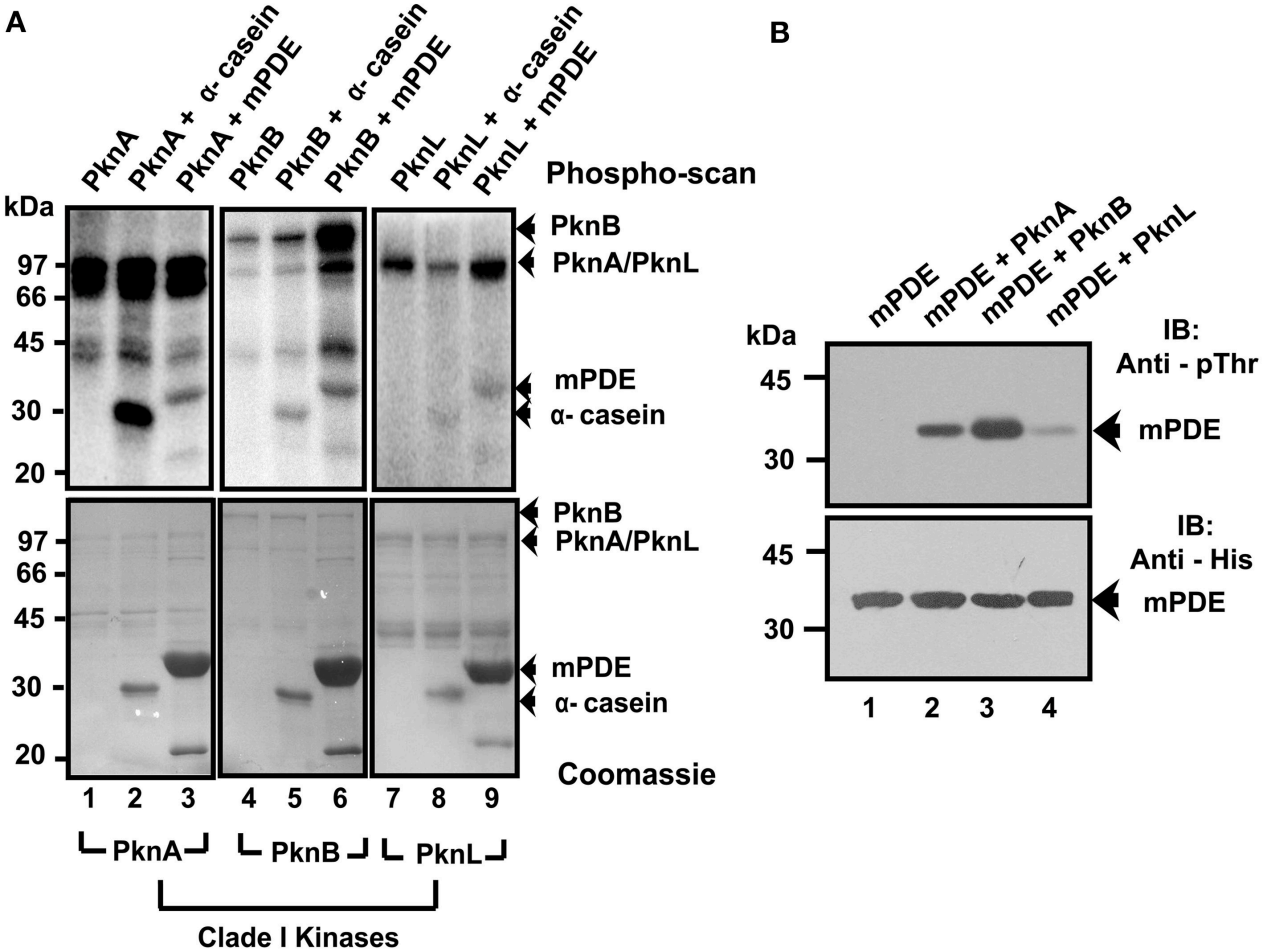
**mPDE is phosphorylated by Clade I mycobacterial Ser/Thr kinases**. **(A)** *In vitro* phosphorylation of Ni-NTA purified mPDE with different mycobacterial kinases belonging to Clade I. Kinase assay utilizing [γ- ^32^P] labeled ATP was performed by incubating MBP-tagged PknA (0.28 μM) or PknB (0.23 μM) or PknL (0.57 μM) with His-tagged mPDE purified from *E. coli* strain BL21(DE3) or α-casein (4 μM, internal control) using protocol mentioned in the text. Reactions were stopped by adding 5X SDS dye followed by heating at 90°C for 5 min. Samples were resolved in 10% SDS-PAGE gel and signals were visualized through a phospho-imaging device. Coomassie stained gels were used as loading control. **(B)** *In vivo* phosphorylation of mPDE with clade I kinases. Recombinant His-tagged mPDE protein was purified using Ni-NTA affinity chromatography of lysates from BL21(DE3) cells expressing either PknA or PknB or PknL in pMAL-c2 vector. Phosphorylation status of the protein(s) was determined in western blot by probing with anti-phosphothreonine antibody (upper panel). Immunoblot (lower panel) using anti-His antibody as the probe served as a loading control.

To elucidate, if transphosphorylation of mPDE is PknA specific, we assessed the ability of other kinases belonging to the same clade (PknB and PknL) for such activities utilizing *in vitro* kinase assay. For this, we used full length PknA and a basic protein like α-casein as internal controls. As shown in Figure 4A, both PknB and PknL, like PknA were able to transfer [γ-^32^P] to either α-casein or mPDE. However, magnitude of transphosphorylation ability of different kinases, as has been reflected in phosphorylated band intensity of the substrates was found to be varied (Figure 4A, compare lanes 2, 5, and 8 for α-casein or 3, 6, and 9 for mPDE). Surprisingly, despite using same amount of kinase (PknA or PknB or PknL) in transphosphorylation assay, the addition of mPDE in reaction mixture always resulted in increased band intensity for phospho-PknB compared to that of the PknB alone (Figure 4A, compare upper and lower panels, lanes 4 and 6). Of note, autorads/phosphoimaging scans with kinases especially in fusion with MBP often exhibited bands at around ~45 kDa besides specific signals and this was consistent with previous reports (Thakur and Chakraborti, 2006; Zhou et al., 2015). These bands could either be phosphorylated protein without fusion tag or degradation product(s) or non-specific entities within the reaction mix. We further validated this observation by immunoblotting of Ni-NTA purified mPDE from *E. coli* BL21(DE3) cell lysates co-expressed with either PknB or PknL using anti-phosphothreonine antibody. As observed with PknA, mPDE protein was recognized by anti-phosphothreonine antibody in all cases (Figure 4B, upper panel; see also Figure 2C, right panel). Ponceau-S stained blot served as a loading control for the same (see Figure S2) and authenticity of the protein was confirmed by probing with anti-His antibody (Figure 4B, lower panel). Interestingly, despite the loading of same amount of mPDE, the phospho-mPDE as reflected in band intensity varied with the kinase used (Figure 4B, compare upper and lower panels). Thus, these findings argued in favor of *in vitro* phosphorylating ability of mPDE by mycobacterial eukaryotic-type Ser/Thr kinases belonging to Clade I.

**Figure 5:**
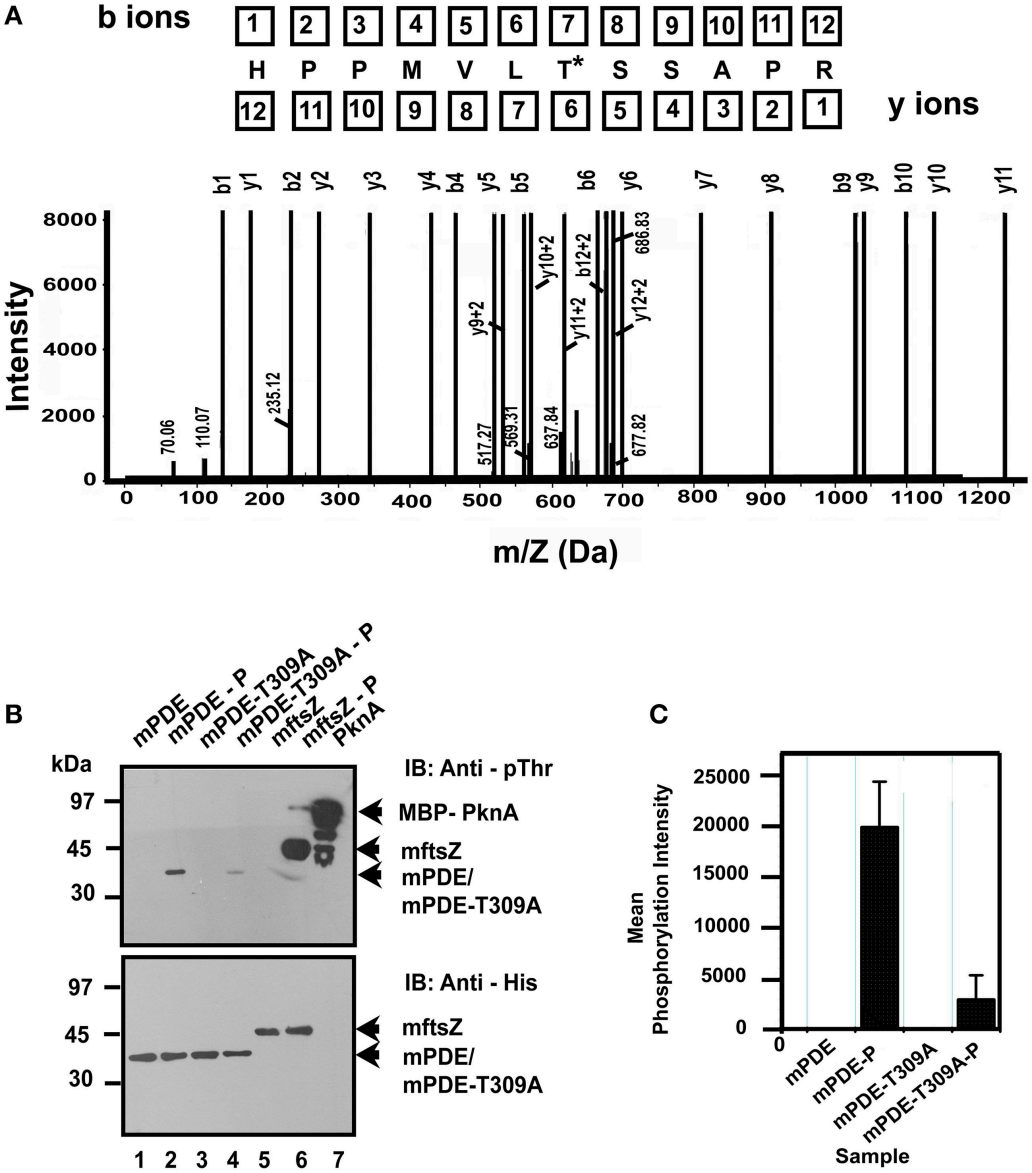
**Identification of Thr-309 as a phosphorylating residue**. **(A)** Identification of phosphorylated residue by LC-MS/MS. Phosphorylated His_6_-mPDE (obtained when co-expressed with pMAL-PknA in BL21(DE3) cells) along with its unphosphorylated counterpart (8 μg each) were hydrolyzed in ammonium bicarbonate buffer at 37°C for 16 h by in-solution trypsin digestion method. Samples were then used for LC-MS/MS analysis. Phospho-site identification was done using protein pilot. Fragmentation spectrum is shown for the modified peptide bearing threonine-309 residue phosphorylated by PknA. Asterisk (^*^) indicates modified Thr residue. **(B)** Thr-309 is predominantly phosphorylated by PknA *in vivo*. mPDE-T309A generated by site directed mutagenesis was tested for any alteration in phosphorylation profile compared to the wild-type in western blotting probing with anti-phosphothreonine antibody (upper panel). The same blot following stripping was probed with anti-His antibody served as loading control (lower panel). mFtsZ and PknA were used as internal controls for the experiment. **(C)** Quantitative analysis to determine the arbitrary mean phosphorylation intensities of mPDE-P and mPDE-T309A-P from western blots were calculated using ImageJ (Abràmoff et al., 2004; Schneider et al., 2012) and plotted utilizing Microcal Origin 5.0 software. Western blot shown is a representation of four independent experiments from three different preparations. Notations used: mPDE-P, phosphorylated mPDE; mPDE-T309A-P, phosphorylated mPDE-T309A.

It was further intriguing to identify the residues of mPDE phosphorylated by PknA. For this purpose, lysate from *E. coli* BL21(DE3) cells co-expressing His_6_-mPDE as well as MBP-PknA was purified through Ni-NTA column, dialyzed, trypsin-digested and finally used for LC-MS/MS analysis along with unphosphorylated protein. Protein pilot and MASCOT search led to the identification of Thr-309 of mPDE as the phosphorylated residue with 99% confidence (Figure 5A; also see Table S1). To confirm this observation, we generated a mutant mPDE-T309A by site directed mutagenesis that replaced threonine with alanine. Immunoblot analysis with anti-phosphothreonine antibody showed that the mutant protein (mPDE-T309A) was less phosphorylated than the wild-type (Figure 5B; compare lanes 2 and 4). His_6_-mFtsZ, which is also a substrate of PknA was used here as a control. Authenticity of different proteins used in this experiment was confirmed by western blotting with anti-His antibody. Quantitation of phosphorylation intensity of bands from western blots recognized by anti-phosphothreonine antibody through ImageJ analysis (in arbitrary units; *n* = 4) revealed ~8-fold decrease in transphosphorylation of mPDE-T309A compared to that of the wild-type mPDE (Figure 5C).

### Phosphorylation of mPDE is associated with its cell wall localization

**Figure 6:**
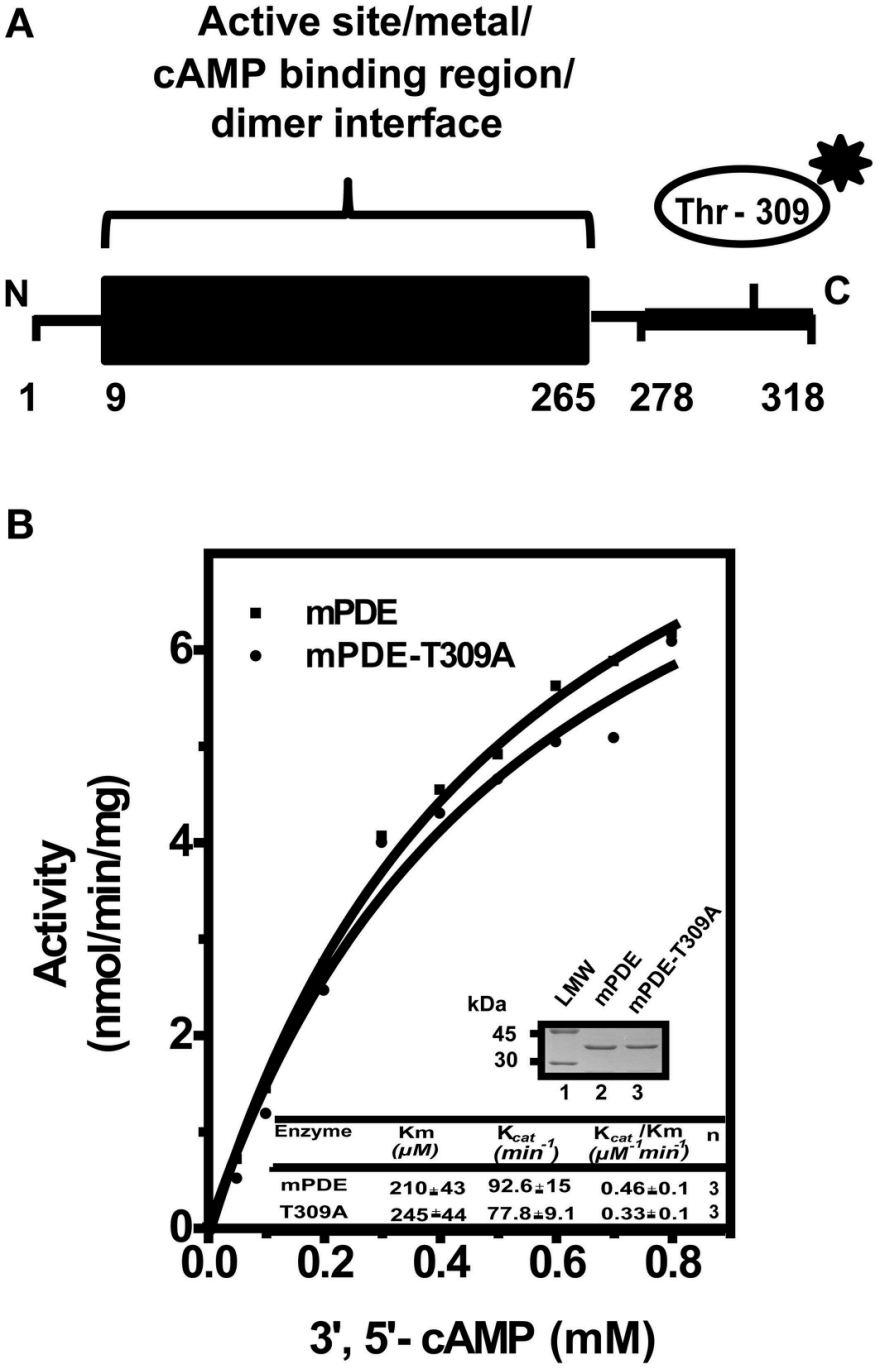
**Comparison of catalytic activities of mPDE and mPDE-T309A**. **(A)** Schematic representation of domain architecture of mPDE. Position of Thr-309 is indicated by asterisk. N and C denote amino- and carboxy -terminal ends of mPDE respectively. **(B)** Kinetic analysis. His_6_-mPDE and His_6_-mPDE-T309A proteins were purified through Ni-NTA columns. Commercially available PDE assay kit was used to determine kinetic parameters of mPDE and mPDE-T309A proteins as mentioned under “Materials and Methods.” Insets show Coomassie stained gel with purified wild-type (mPDE) and mutant (mPDE-T309A) proteins (3 μg/lane) used for determining catalytic parameters (table). Notation used: LMW, low molecular weight marker.

The crystal structure of mPDE revealed that the active site comprising of metal and cAMP-binding sites are confined within N-terminal 265 amino acid residues (Shenoy et al., 2007; Podobnik et al., 2009). The C-terminal 20 amino acids, on the other hand, is disordered as no electron density was observed for this region (Podobnik et al., 2009; Matange et al., 2014). Available reports indicated that the C-terminal region has patches, which support protein localization to cell wall (Podobnik et al., 2009; Matange et al., 2014). Thus, the presence of phosphorylating Thr-309 residue (Figure 6A) fascinated us in evaluating the role of phosphorylation on the protein. Kinetic analysis of enzyme activities of mPDE and mPDE-T309A proteins exhibited a typical Michaelis-Menten curve (Figure 6B) with comparable catalytic (Km and K_cat_) parameters (Inset, Figure 6B).

**Figure 7:**
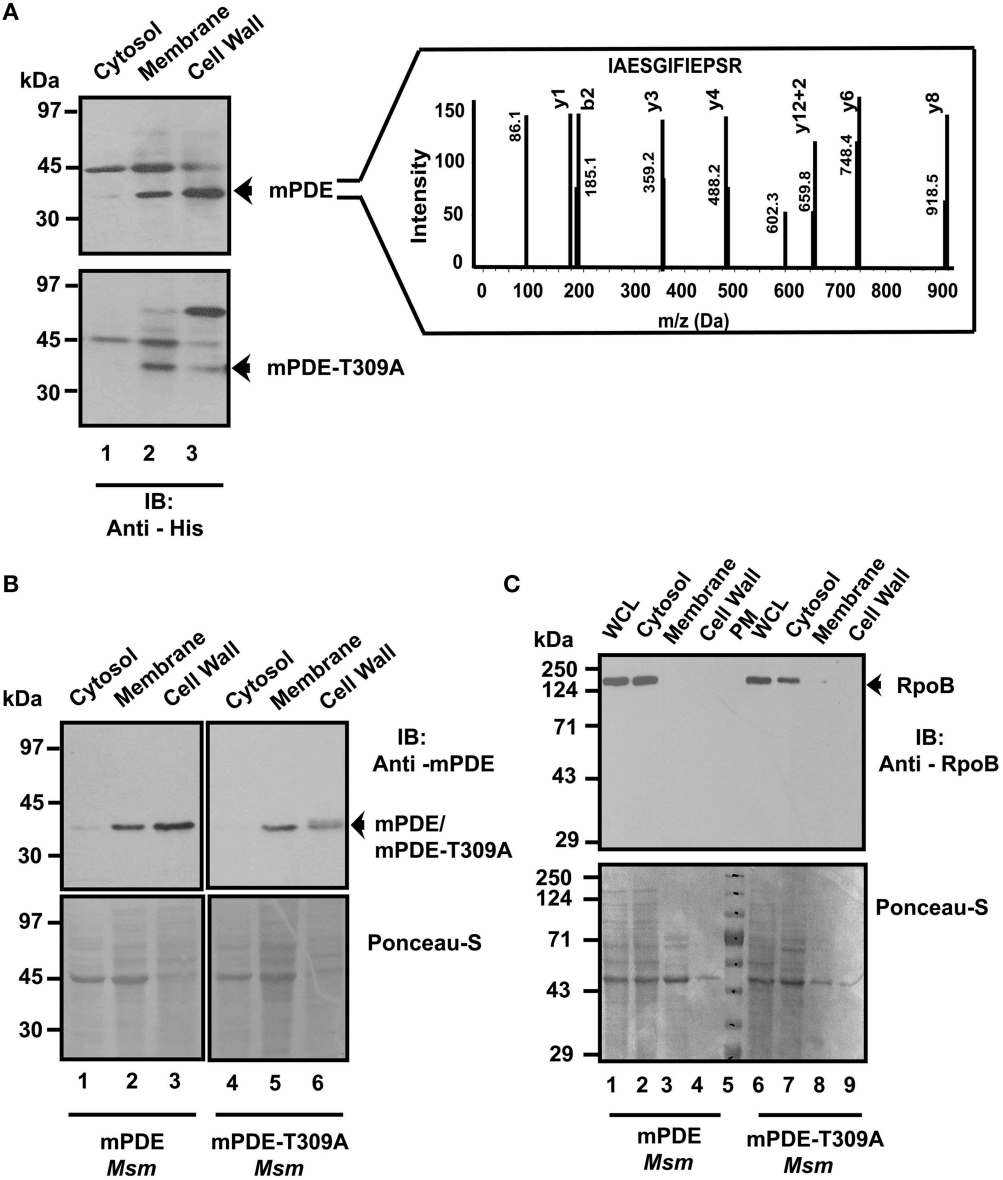
**mPDE phosphorylation affects its cell wall localization**. Phosphorylation influences sub-cellular localization of mPDE. pVV2-mPDE or pVV2-mPDE-T309A were expressed in *M. smegmatis*. Following sub-cellular fractionation, samples (10 μg protein/slot) were resolved in 10% SDS-PAGE and subjected to immunoblotting. **(A)** Western Blotting with anti-His antibody. Upper and lower panels represent *M. smegmatis* sub-cellular fractions expressing wild-type and mPDE-T309A proteins probed with anti-His antibody. Ponceau-S staining of this blot served as a loading control (see Figure S3). Inset represents the identity of mPDE protein in *M. smegmatis* cell wall fraction through in-gel trypsin digestion followed by LC-MS/MS and analyzed by protein pilot software. **(B)** Western blotting with anti-mPDE antibody to confirm sub-cellular localization of wild-type and mPDE-T309A proteins following expression in *M. smegmatis*. Reproducibility of western blot results was verified atleast three times from two different protein preparations. Numbers indicate molecular weight markers in kDa. **(C)** Sub-cellular fractions did not show any cross contamination. Upper panel indicates western blotting of cellular fractions of *M. smegmatis* expressing mPDE/mPDE-T309A utilizing anti-RpoB antibody to probe localization of cytosolic marker RpoB in cells. Numbers indicate pre-stained molecular weight markers in kDa. Lower panels of **(B,C)** depict Ponceau-S stained blots as loading controls for the experiments. Notations used: WCL, whole cell lysate; *Msm, M. smegmatis;* PM, pre-stained marker.

We further expressed both His_6_-mPDE and His_6_-mPDE-T309A in *M. smegmatis*, prepared sub-cellular fractions (cytosol, membrane, and cell wall) and compared protein profiles in western blotting using anti-His or anti-mPDE antibody. Although probing the protein with anti-His antibody yielded non-specific bands, we observed band of expected molecular mass of mPDE at membrane and cell wall fractions (Figure 7A top and bottom panels, lanes 2 and 3). Such non-specific band(s) is not unusual considering the fact that the cellular fractions might possess several proteins bearing stretch of histidine residues, which would have been recognized by anti-His antibody. The intensity of the band, however, at the expected molecular size of mPDE in western blotting was more in cell wall compared to that of the membrane fraction (Figure 7A, compare lanes 2 and 3 of upper and lower panels). Comparative analysis on the localization of mPDE-T309A vs. mPDE revealed that there was no significant alteration in the distribution pattern of the protein in membrane, but its intensity was significantly lower in cell wall fraction (Figure 7A, see lanes 2 and 3 of top and bottom panels). Considering the unaltered intensity of the non-specific band at ~70 kDa, variation seen in Figure 7A is independent of expression artifact or loading defect in SDS-PAGE gel. Subsequent mass spectrometry experimental results indicated that the ~70 kDa band seem to arise from a mixture of proteins (GroEL/GntR family transcription regulator/phosphoglycerate mutase/glyceraldehyde-3-phosphate dehydrogenase etc.) but not mPDE (please see Table S2). In fact, these proteins were classically considered to be restricted to cell interiors but have later also shown to be cell surface/cell wall associated [please see http://tuberculist.epfl.ch/ and references (Mawuenyega et al., 2005; He and De Buck, 2010; Boradia et al., 2014)]. However, intensity of this band varied from preparation to preparation despite loading of the same amount of protein. The identity of mPDE in *M. smegmatis* cell wall was also confirmed by mass spectrometry (inset, Figure 7A; also see Table S3). To validate our findings, we monitored the localization of mPDE/mPDE-T309A in sub-cellular fractions using anti-mPDE antibody following their overexpression in *M. smegmatis*. Our findings clearly demonstrated that mutation of phosphorylating residue (Thr-309) to alanine affected the localization of protein to cell wall (Figure 7B, compare lanes 3 and 6 of upper panel), whereas the presence of other non-specific bands obtained in Figure 7A were not seen with mPDE specific antibody. Western blotting using anti-RpoB antibody [predominantly a cytosolic marker, see reference (Chao et al., 2013)] was performed to rule out the possibility of cross-contamination between cellular fractions prepared (Figure 7C, upper panel).

**Figure 8:**
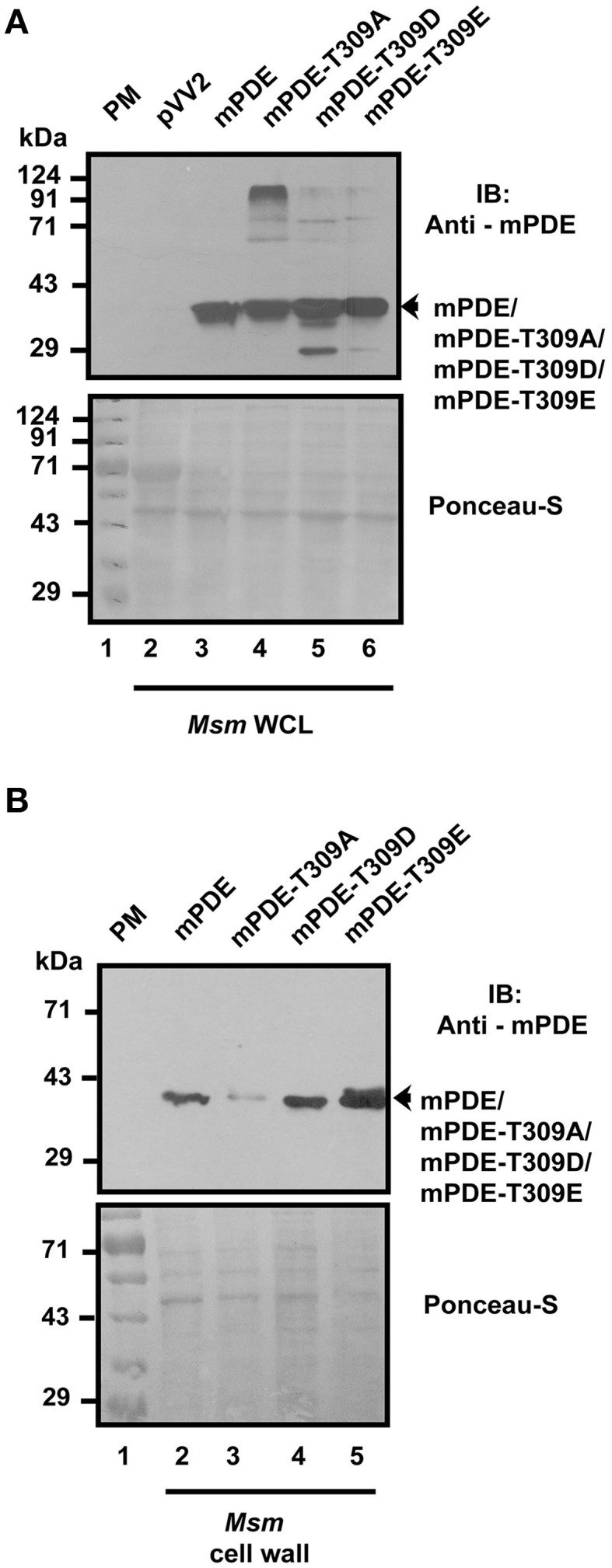
**Cell wall localization of phosphomimics of mPDE. (A)** Monitoring protein expression levels in *M. smegmatis* whole cell extracts. Whole cell lysates of *M. smegmatis* (10 μg/slot, upper panel) expressing either wild-type or phosphoablative or phosphomimetic mutants were resolved in 10% SDS-PAGE gel and subjected to immunoblotting utilizing anti-mPDE antibody. **(B)** Phosphomimetic mutants localizes similar to wild-type. Cell wall fraction (10 μg/slot) isolated from *M. smegmatis* cells expressing mPDE or mPDE-T309A/D/E was probed with anti-mPDE antibody (lower panel). Ponceau-S stained blot represents loading control and numbers indicate molecular mass of pre-stained molecular weight marker used in this study. Notations used: WCL, whole cell lysate; *Msm, M. smegmatis*; PM, pre-stained marker.

Available literature suggested that phosphorylation renders negative charge to a protein, which in turn influences its functionality (Wagner et al., 2004). To have an insight on this aspect, we compared protein localization profiles of wild-type, mPDE-T309A mutant, and phosphomimics (mPDE-T309D and mPDE-T309E) in cell wall fractions following their expression in *M. smegmatis* (Figure 8A, upper panel). Western blotting using anti-mPDE antibody revealed that cell wall localization of the protein was affected only in mPDE-T309A while both the phosphomimics behaved like wild-type mPDE (Figure 8B). Thus, our results established that eukaryotic-type Ser/Thr kinase mediated phosphorylation of mPDE at C-terminal Thr-309 residue portrayed negative charge to the protein, which in turn endorsed its cell wall localization.

### Thr-309 is conserved in *M. tuberculosis* complex

**Figure 9:**
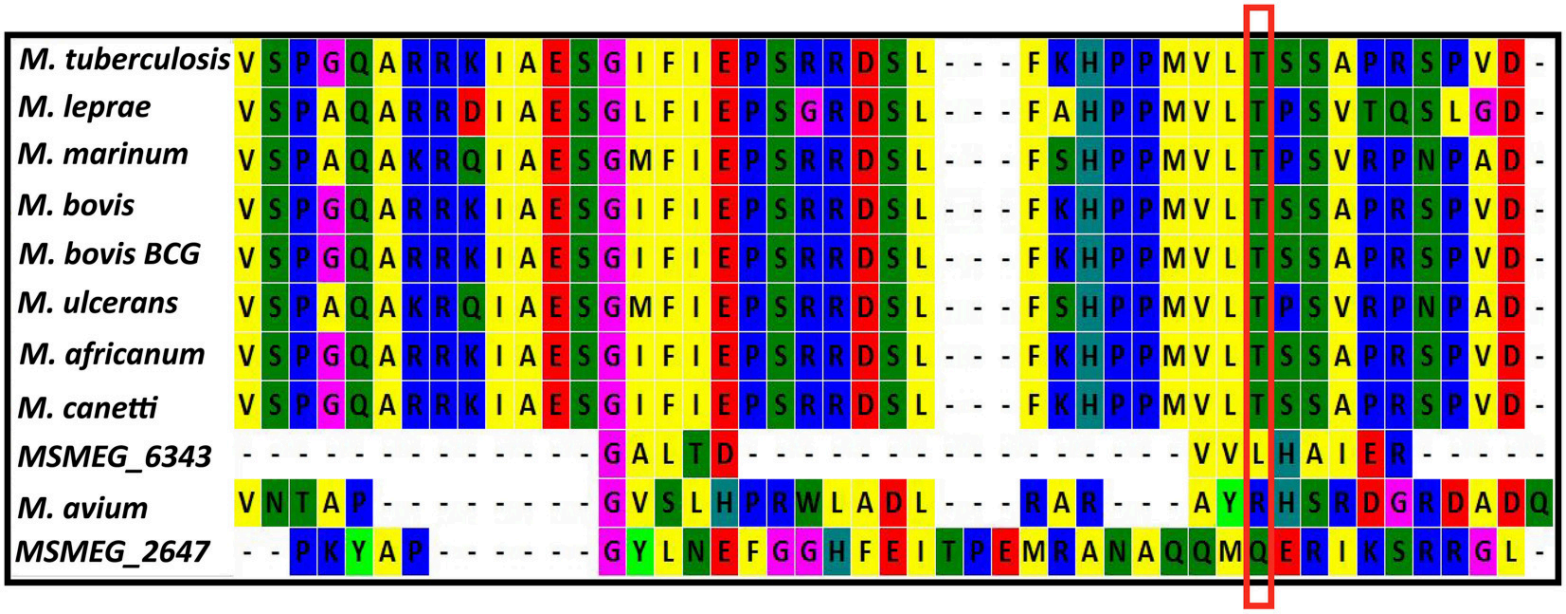
**Thr-309 is conserved in mPDE orthologs**. Multiple sequence alignment of phosphodiesterase was performed between different mycobacterial species. Reciprocal best blast hits of mPDE were obtained from MycoRRdb; manual comparative genomics was done to obtain phosphodiesterase from *M. smegmatis* and *M. avium*; protein sequences were obtained from NCBI; alignment was done by Mega 6.0 software using Muscle algorithm. C-termini of these sequences are displayed in the figure and conserved threonine is enclosed within red rectangular box.

We further considered reciprocal best blast hits for mPDE using MycoRRdb database (Midha et al., 2012) and protein sequences for those whose functional annotation has been done were procured from NCBI (ncbi.nlm.nih.gov). Multiple sequence alignment revealed a high sequence identity between the mPDE orthologs in different mycobacterial species. Interestingly, Thr-309 residue of mPDE is conserved in all the orthologs (Figure 9; PDE sequences from *M. smegmatis* and *M. avium* were used as outgroups), illustrating its probable evolutionary significance.

## Discussion

Signal transduction in mycobacteria involves highly efficient post translational modification machinery and secondary messengers. Cyclic AMP is one such secondary messenger, which is produced in an exceptionally high amount compared to other bacteria (Shenoy and Visweswariah, 2006). However, only single phosphodiesterase (mPDE) is identified and characterized, which regulates mycobacterial cAMP levels within cells (Shenoy et al., 2005). Interestingly, its overexpression led to reduction in 3′, 5′- cAMP level in *E. coli* or *M. smegmatis* by 30%, while it is of 50% in the case of *M. tuberculosis* indicating a negative feedback control is operational (Agarwal et al., 2009). Thus, existence of an equilibrium between the levels of cellular cAMP synthesized and degraded or exported out of the cells has already been postulated (Bai et al., 2011). Apart from cAMP hydrolysis, mPDE also plays a key role in maintaining cell wall permeability (Podobnik et al., 2009; Matange et al., 2014) and regulating downstream signaling independent of that mediated by cAMP receptor protein (Matange et al., 2013). Similarly, a well-orchestrated post translational machinery involving cascade of events through phosphorylation and dephosphorylation is mediated by 11 eukaryotic-type Ser/Thr kinases and a cognate phosphatase in *M. tuberculosis* which have been identified to regulate plethora of cellular functions (Molle et al., 2006; Molle and Kremer, 2010; Pereira et al., 2011). Accordingly, in the current study we assessed the ability of mycobacterial kinases belonging to clade I (PknA/PknB/PknL) to transphosphorylate mPDE. Since, mPDE is phosphorylated by all these kinases with varying degree, our results shown in Figures 2, 4, offer first clue for it being a probable substrate of mycobacterial eukaryotic-type Ser/Thr kinases. Furthermore, mPDE is not a basic protein (calculated pI = 6.1) so that it would be non-specifically phosphorylated by these kinases. At this juncture, it needs to be emphasized here that recent genome-wide phosphoproteome mapping of *M. tuberculosis* did not identify mPDE as a phosphorylated protein (Prisic et al., 2010; Prisic and Husson, 2014). However, this is not unlikely considering the fact that experimental conditions might influence the results of mass spectrometric data.

Like eukaryotes, where phosphorylation is one of the important mechanisms to regulate phosphodiesterase activity as well as its functionality (Omori and Kotera, 2007), such a control has not yet been reported in prokaryotes. In this scenario, our results argue that phosphorylation mediated regulation is not confined to eukaryotes only. Interestingly, single threonine residue (Thr-309) at extreme C-terminus of the protein was predominantly phosphorylated with a significant number of modified b and y ions generated in our mass spectrometric study (Figure 5A). Moreover, when this residue was mutated to alanine and co-expressed along with PknA in *E. coli* BL21(DE3) cells, mPDE-T309A was rarely recognized by anti-phosphothreonine antibody (Figure 5B). This highlights the importance of Thr-309 as the phosphorylating residue. Conversely, mPDE-T309A protein did not show any significant alteration in its enzymatic activity compared to that of the wild-type mPDE (Figure 6B), which did not seem to be inconsistent considering the mutation at a non-catalytic site. Thus, in the absence of any Ser/Thr phosphatase use of *E. coli* system as a surrogate host for the expression of mycobacterial Ser/Thr kinases aided us in studying occurrence of phosphorylation of mPDE.

We further introduced *M. tuberculosis* phosphodiesterase gene under the control of an inducible promoter in non-pathogenic saprophyte *M. smegmatis*, where intrinsic kinases such as PknA, PknB and PknL are present but there is no close orthologs of mPDE (Etienne et al., 2005; Narayan et al., 2007; Podobnik et al., 2009). Monitoring the phosphorylation status of the mPDE in such a scenario revealed it as a phospho-protein (Figure 3), though the intensity of phospho-signal was quite low compared to the protein purified from *E. coli* BL21(DE3) cells (Figures 2, 4). This might be an effect of phosphatase present in *M. smegmatis* along with multiple eukaryotic-type Ser/Thr kinases. Surprisingly, we noticed an alteration between wild-type and mPDE-T309A while monitoring sub-cellular localization pattern of these proteins (Figures 7A,B, 8B). On the other hand, Thr-309 when substituted with phosphomimics (Asp or Glu) behaved similar to that of the wild-type (Figure 8B). Such an observation illustrated the importance of the negative charge of the protein (rendered by its phosphorylation) in cell wall anchorage. Our findings are consistent with a previous report demonstrating that phosphorylation at Thr-16 of PDE10A in humans is necessary for its localization from membrane to cytosol (Kotera et al., 2004; Charych et al., 2010). It is therefore logical to infer that phosphorylation mediated control of phosphodiesterases as evident in eukaryotes is also a reality in mPDE despite distinction in their phylogenetic placement (Figure 1). Furthermore, alignment of mPDE with other mycobacterial PDE sequences also explicates Thr-309 as a conserved entity in all the orthologs within *M. tuberculosis* complex (Figure 9) suggesting its phosphorylation to be an evolutionarily important phenomenon. Alternatively, the presence of proline or serine at +1 position of Thr-309 of mPDE ortholgs (Figure 9) might be crucial to maintain the structural dynamicity, thereby governing phosphorylation (at Thr-309) in these proteins. Unraveling these aspects of mycobacterial signal transduction mechanisms will therefore open up the new horizon towards understanding of *M. tuberculosis* basic biology.

## Conclusion

Our results established that phosphorylation of mycobacterial phosphodiesterase, which is mediated by eukaryotic-type Ser/Thr kinases like PknA, leads to its cell wall localization. Thus, this finding indeed insinuates the probable functional interplay between two distinct signaling cascades, one catalyzing post translational modification, and the other involving small molecule trafficking. Further studies in this direction would certainly be expedient to develop potential anti-mycobacterial compounds in future.

## Author contributions

PC and NM conceived the idea; planned experiments, analyzed the results and wrote the manuscript; NM carried out experiments.

### Conflict of interest statement

The authors declare that the research was conducted in the absence of any commercial or financial relationships that could be construed as a potential conflict of interest.

## Abbreviations

cAMP: 3′, 5′- cyclic adenosine monophosphate
mPDE: *M. tuberculosis* phosphodiesterase
MBP: Maltose Binding Protein
His_6_: 6X Histidine tag
OADC: Oleic acid albumin dextrose catalase growth enrichment
LC-MS/MS: Liquid chromatography coupled tandem mass spectrometry
*Msm*: *M. smegmatis*.

## References

[B1] Abràmoff M. D. Magalhães P. J. Ram S. J. (2004). Image processing with ImageJ. Biophotonics Int. 11, 36–42.

[B2] Agarwal N. Lamichhane G. Gupta R. Nolan S. Bishai W. R. (2009). Cyclic AMP intoxication of macrophages by a *Mycobacterium tuberculosis* adenylate cyclase. Nature 460, 98–102. 10.1038/nature08123 19516256

[B3] Bai G. Knapp G. S. McDonough K. A. (2011). Cyclic AMP signalling in mycobacteria: redirecting the conversation with a common currency. Cell Microbiol. 13, 349–358. 10.1111/j.1462-5822.2010.01562.x 21199259 PMC3785248

[B4] Baronian G. G. Ginda K. Berry L. Cohen-Gonsaud M. Zakrzewska-CzerwiÅ J. Jakimowicz D.. (2015). Phosphorylation of *Mycobacterium tuberculosis* ParB participates in regulating the ParABS chromosome segregation system. PLoS ONE 10:e0119907. 10.1371/journal.pone.0119907 25807382 PMC4373775

[B5] Bessay E. P. Blount M. A. Zoraghi R. Beasley A. Grimes K. A. Francis S. H.. (2008). Phosphorylation increases affinity of the phosphodiesterase-5 catalytic site for tadalafil. J. Pharmacol. Exp. Ther. 325, 62–68. 10.1124/jpet.107.133405 18199808

[B6] Boradia V. M. Malhotra H. Thakkar J. S. Tillu V. A. Vuppala B. Patil P.. (2014). *Mycobacterium tuberculosis* acquires iron by cell-surface sequestration and internalization of human holo-transferrin. Nat. Commun. 5:4730. 10.1038/ncomms5730 25163484

[B7] Chaba R. Raje M. Chakraborti P. K. (2002). Evidence that a eukaryotic-type serine/threonine protein kinase from *Mycobacterium tuberculosis* regulates morphological changes associated with cell division. Eur. J. Biochemi. 269, 1078–1085. 10.1046/j.1432-1033.2002.02778.x 11856348

[B8] Chakraborti P. K. Matange N. Nandicoori V. K. Singh Y. Tyagi J. S. Visweswariah S. S. (2011). Signalling mechanisms in Mycobacteria. Tuberculosis 91, 432–440. 10.1016/j.tube.2011.04.005 21570916

[B9] Chao M. C. Kieser K. J. Minami S. Mavrici D. Aldridge B. B. Fortune S. M.. (2013). Protein complexes and proteolytic activation of the cell wall hydrolase RipA regulate septal resolution in mycobacteria. PLoS Pathog. 9:e1003197. 10.1371/journal.ppat.1003197 23468634 PMC3585148

[B10] Charych E. I. Jiang L.-X. Lo F. Sullivan K. Brandon N. J. (2010). Interplay of palmitoylation and phosphorylation in the trafficking and localization of phosphodiesterase 10A: implications for the treatment of schizophrenia. J. Neurosci. 30, 9027–9037. 10.1523/JNEUROSCI.1635-10.2010 20610737 PMC6632485

[B11] Chevalier F. L. Cascioferro A. Majlessi L. Herrmann J. L. Brosch R. (2014). *Mycobacterium tuberculosis* evolutionary pathogenesis and its putative impact on drug development. Future Microbiol. 9, 969–985. 10.2217/fmb.14.70 25302954

[B12] Cole S. Brosch R. Parkhill J. Garnier T. Churcher C. Harris D.. (1998). Deciphering the biology of *Mycobacterium tuberculosis* from the complete genome sequence. Nature 393, 537–544. 10.1038/31159 9634230

[B13] Dar H. Chakraborti P. (2010). Intermolecular phosphotransfer is crucial for efficient catalytic activity of nucleoside diphosphate kinase. Biochem. J. 430, 539–549. 10.1042/BJ20100026 20575762

[B14] Dave J. A. Van Pittius N. C. G. Beyers A. D. Ehlers M. R. W. Brown G. D. (2002). Mycosin-1, a subtilisin-like serine protease of *Mycobacterium tuberculosis*, is cell wall-associated and expressed during infection of macrophages. BMC Microbiol. 2:30. 10.1186/1471-2180-2-30 12366866 PMC131053

[B15] Dey B. Bishai W. R. (2014). Crosstalk between *Mycobacterium tuberculosis* and the host cell. Semin. Immunol. 26, 486–496. 10.1016/j.smim.2014.09.002 25303934 PMC4250340

[B16] Dhiman R. K. Schulbach M. C. Mahapatra S. Baulard A. R. Vissa V. Brennan P. J.. (2004). Identification of a novel class of ω,E,E-farnesyl diphosphate synthase from *Mycobacterium tuberculosis*. J. Lipid Res. 45, 1140–1147. 10.1194/jlr.M400047-JLR200 15060088

[B17] Edgar R. C. (2004). MUSCLE: multiple sequence alignment with high accuracy and high throughput. Nucleic Acids Res. 32, 1792–1797. 10.1093/nar/gkh340 15034147 PMC390337

[B18] Etienne G. Laval F. Villeneuve C. Dinadayala P. Abouwarda A. Zerbib D.. (2005). The cell envelope structure and properties of *Mycobacterium smegmatis* mc2155: is there a clue for the unique transformability of the strain? Microbiology 151, 2075–2086. 10.1099/mic.0.27869-0 15942014

[B19] Gibbons H. S. Wolschendorf F. Abshire M. Niederweis M. Braunstein M. (2007). Identification of two *Mycobacterium smegmatis* lipoproteins exported by a SecA2-dependent pathway. J. Bacteriol. 189, 5090–5100. 10.1128/JB.00163-07 17496088 PMC1951849

[B20] He Z. De Buck J. (2010). Localization of proteins in the cell wall of Mycobacterium avium subsp. paratuberculosis K10 by proteomic analysis. Proteome Sci. 8:21. 10.1186/1477-5956-8-21 20377898 PMC2859856

[B21] Ho S. N. Hunt H. D. Horton R. M. Pullen J. K. Pease L. R. (1989). Site-directed mutagenesis by overlap extension using the polymerase chain reaction. Gene 77, 51–59. 10.1016/0378-1119(89)90358-2 2744487

[B22] Hong Y. Zhou X. Fang H. Yu D. Li C. Sun B. (2013). Cyclic di-GMP mediates *Mycobacterium tuberculosis* dormancy and pathogenecity. Tuberculosis 93, 625–634. 10.1016/j.tube.2013.09.002 24080120

[B23] Kang C.-M. Abbott D. W. Park S. T. Dascher C. C. Cantley L. C. Husson R. N. (2005). The *Mycobacterium tuberculosis* serine/threonine kinases PknA and PknB: substrate identification and regulation of cell shape. Genes Dev. 19, 1692–1704. 10.1101/gad.1311105 15985609 PMC1176007

[B24] Kitamura T. Kitamura Y. Kuroda S. Hino Y. Ando M. Kotani K.. (1999). Insulin-induced phosphorylation and activation of cyclic nucleotide phosphodiesterase 3B by the serine-threonine kinase Akt. Mol. Cell. Biol. 19, 6286–6296. 10.1128/MCB.19.9.6286 10454575 PMC84592

[B25] Kotera J. Sasaki T. Kobayashi T. Fujishige K. Yamashita Y. Omori K. (2004). Subcellular localization of cyclic nucleotide phosphodiesterase type 10A variants, and alteration of the localization by cAMP-dependent protein kinase-dependent phosphorylation. J. Biol. Chem. 279, 4366–4375. 10.1074/jbc.M308471200 14604994

[B26] Lakshminarayan H. Narayanan S. Bach H. Sundaram K. G. P. Av-Gay Y. (2008). Molecular cloning and biochemical characterization of a serine threonine protein kinase, PknL, from *Mycobacterium tuberculosis*. Protein Expr. Purif. 58, 309–317. 10.1016/j.pep.2007.12.012 18276158

[B27] Lindh R. Ahmad F. ResjÃ S. James P. Yang J. S. Fales H. M.. (2007). Multisite phosphorylation of adipocyte and hepatocyte phosphodiesterase 3B. Biochim. Biophys. Acta 1773, 584–592. 10.1016/j.bbamcr.2007.01.010 17320989

[B28] Liu H. Maurice D. H. (1999). Phosphorylation-mediated Activation and translocation of the cyclic AMP-specific phosphodiesterase PDE4D3 by cyclic AMP-dependent protein kinase and mitogen-activated protein kinases a potential mechanism allowing for the coordinated regulation of PDE4D Activity and Targeting. J. Biol. Chem. 274, 10557–10565. 10.1074/jbc.274.15.10557 10187850

[B29] Macphee C. H. Reifsnyder D. H. Moore T. A. Lerea K. M. Beavo J. A. (1988). Phosphorylation results in activation of a cAMP phosphodiesterase in human platelets. J. Biol. Chem. 263, 10353–10358. 2839485

[B30] Manikandan K. Sabareesh V. Singh N. Saigal K. Mechold U. Sinha K. M. (2014). Two-step synthesis and hydrolysis of cyclic di-AMP in *Mycobacterium tuberculosis*. PLoS ONE 9:e86096. 10.1371/journal.pone.0086096 24465894 PMC3900455

[B31] Matange N. Hunt D. M. Buxton R. S. Visweswariah S. S. (2013). Overexpression of the Rv0805 phosphodiesterase elicits a cAMP-independent transcriptional response. Tuberculosis 93, 492–500. 10.1016/j.tube.2013.05.004 23835087 PMC3776917

[B32] Matange N. Podobnik M. Visweswariah S. S. (2014). The non-catalytic cap domain of a mycobacterial metallophosphoesterase regulates its expression and localization in the cell. J. Biol. Chem. 289, 22470–22481. 10.1074/jbc.M114.578328 24970891 PMC4139253

[B33] Mawuenyega K. G. Forst C. V. Dobos K. M. Belisle J. T. Chen J. Bradbury E. M.. (2005). *Mycobacterium tuberculosis* functional network analysis by global subcellular protein profiling. Mol. Biol. Cell 16, 396–404. 10.1091/mbc.E04-04-0329 15525680 PMC539182

[B34] McDonough K. A. Rodriguez A. (2012). The myriad roles of cyclic AMP in microbial pathogens: from signal to sword. Nat. Rev. Microbiol. 10, 27–38. 10.1038/nrmicro2688 22080930 PMC3785115

[B35] Midha M. Prasad N. K. Vindal V. (2012). MycoRRdb: a database of computationally identified regulatory regions within intergenic sequences in mycobacterial genomes. PLoS ONE 7:e36094. 10.1371/journal.pone.0036094 22563442 PMC3338573

[B36] Molle V. Kremer L. (2010). Division and cell envelope regulation by Ser/Thr phosphorylation: mycobacterium shows the way. Mol. Microbiol. 75, 1064–1077. 10.1111/j.1365-2958.2009.07041.x 20487298

[B37] Molle V. Zanella-Cleon I. Robin J. P. Mallejac S. Cozzone A. J. Becchi M. (2006). Characterization of the phosphorylation sites of *Mycobacterium tuberculosis* serine/threonine protein kinases, PknA, PknD, PknE, and PknH by mass spectrometry. Proteomics 6, 3754–3766. 10.1002/pmic.200500900 16739134

[B38] Narayan A. Sachdeva P. Sharma K. Saini A. K. Tyagi A. K. Singh Y. (2007). Serine threonine protein kinases of mycobacterial genus: phylogeny to function. Physiol. Genomics 29, 66–75. 10.1152/physiolgenomics.00221.2006 17148687

[B39] Omori K. Kotera J. (2007). Overview of PDEs and their regulation. Circ. Res. 100, 309–327. 10.1161/01.RES.0000256354.95791.f1 17307970

[B40] Orme I. M. (2014). Tuberculosis vaccines: types and timings. Clin. Vaccine Immunol. 22, 249–257. 10.1128/CVI.00718-14 25540272 PMC4340897

[B41] Pereira S. F. F. Goss L. Dworkin J. (2011). Eukaryote-like serine/threonine kinases and phosphatases in bacteria. Microbiol. Mol. Biol. Rev. 75, 192–212. 10.1128/MMBR.00042-10 21372323 PMC3063355

[B42] Podobnik M. Tyagi R. Matange N. Dermol U. K. Gupta A. K. Mattoo R.. (2009). A mycobacterial cyclic AMP phosphodiesterase that moonlights as a modifier of cell wall permeability. J. Biol. Chem. 284, 32846–32857. 10.1074/jbc.M109.049635 19801656 PMC2781701

[B43] Prisic S. Dankwa S. Schwartz D. Chou M. F. Locasale J. W. Kang C.-M.. (2010). Extensive phosphorylation with overlapping specificity by *Mycobacterium tuberculosis* serine/threonine protein kinases. Proc. Natl. Acad. Sci. U.S.A. 107, 7521–7526. 10.1073/pnas.0913482107 20368441 PMC2867705

[B44] Prisic S. Husson R. N. (2014). *Mycobacterium tuberculosis* serine/threonine protein kinases. Microbiol. Spectrum 2, 1–42. 10.1128/microbiolspec.MGM2-0006-2013 25429354 PMC4242435

[B45] Ravala S. K. Singh S. Yadav G. S. Kumar S. Karthikeyan S. Chakraborti P. K. (2015). Evidence that phosphorylation of threonine in the GT motif triggers activation of PknA, a eukaryotic-type serine/threonine kinase from *Mycobacterium tuberculosis*. FEBS J. 282, 1419–1431. 10.1111/febs.13230 25665034

[B46] Sarin J. Aggarwal S. Chaba R. Varshney G. C. Chakraborti P. K. (2001). B-subunit of phosphate-specific transporter from *Mycobacterium tuberculosis* is a thermostable ATPase. J. Biol. Chem. 276, 44590–44597. 10.1074/jbc.M105401200 11567022

[B47] Schneider C. A. Rasband W. S. Eliceiri K. W. (2012). NIH Image to ImageJ: 25 years of image analysis. Nat. Methods 9, 671–675. 10.1038/nmeth.2089 22930834 PMC5554542

[B48] Shenoy A. R. Capuder M. DraÅ¡kovic P. Lamba D. Visweswariah S. S. Podobnik M. (2007). Structural and biochemical analysis of the Rv0805 cyclic nucleotide phosphodiesterase from *Mycobacterium tuberculosis*. J. Mol. Biol. 365, 211–225. 10.1016/j.jmb.2006.10.005 17059828

[B49] Shenoy A. R. Sreenath N. Podobnik M. Kovacevic M. Visweswariah S. S. (2005). The Rv0805 gene from *Mycobacterium tuberculosis* encodes a 3′, 5′-cyclic nucleotide phosphodiesterase: biochemical and mutational analysis. Biochemistry 44, 15695–15704. 10.1021/bi0512391 16313172

[B50] Shenoy A. R. Visweswariah S. S. (2006). New messages from old messengers: cAMP and mycobacteria. Trends Microbiol. 14, 543–550. 10.1016/j.tim.2006.10.005 17055275

[B51] Tamura K. Stecher G. Peterson D. Filipski A. Kumar S. (2013). MEGA6: molecular evolutionary genetics analysis version 6.0. Mol. Biol. Evol. 30, 2725–2729. 10.1093/molbev/mst197 24132122 PMC3840312

[B52] Thakur M. Chaba R. Mondal A. K. Chakraborti P. K. (2008). Interdomain interaction reconstitutes the functionality of PknA, a eukaryotic type Ser/Thr kinase from *Mycobacterium tuberculosis*. J. Biol. Chem. 283, 8023–8033. 10.1074/jbc.M707535200 18199749

[B53] Thakur M. Chakraborti P. (2008). Ability of PknA, a mycobacterial eukaryotic-type serine/threonine kinase, to transphosphorylate MurD, a ligase involved in the process of peptidoglycan biosynthesis. Biochem. J. 415, 27–33. 10.1042/BJ20080234 18557704

[B54] Thakur M. Chakraborti P. K. (2006). GTPase activity of mycobacterial FtsZ is impaired due to its transphosphorylation by the eukaryotic-type Ser/Thr kinase, PknA. J. Biol. Chem. 281, 40107–40113. 10.1074/jbc.M607216200 17068335

[B55] Wagner L. E. Li W.-H. Joseph S. K. Yule D. I. (2004). Functional consequences of phosphomimetic mutations at key cAMP-dependent protein kinase phosphorylation sites in the type 1 inositol 1, 4, 5-trisphosphate receptor. J. Biol. Chem. 279, 46242–46252. 10.1074/jbc.M405849200 15308649

[B56] Wang L. Slayden R. A. Barry C. E. Liu J. (2000). Cell wall structure of a mutant of *Mycobacterium smegmatis* defective in the biosynthesis of mycolic acids. J. Biol. Chem. 275, 7224–7229. 10.1074/jbc.275.10.7224 10702292

[B57] Yang J. Bai Y. Zhang Y. Gabrielle V. D. Jin L. Bai G. (2014). Deletion of the cyclic di-AMP phosphodiesterase gene (cnpB) in *Mycobacterium tuberculosis* leads to reduced virulence in a mouse model of infection. Mol. Microbiol. 93, 65–79. 10.1111/mmi.12641 24806618 PMC4088933

[B58] Yang W. Zhang L. Lu Z. Tao W. Zhai Z. (2001). A new method for protein coexpression in Escherichia coli using two incompatible plasmids. Protein Expr. Purif. 22, 472–478. 10.1006/prep.2001.1453 11483011

[B59] Zaveri A. Visweswariah S. S. (2013). Cyclic AMP in Mycobacteria: the second messenger comes first. Curr. Sci. 105, 666–675.

[B60] Zhou P. Wong D. Li W. Xie J. Av-Gay Y. (2015). Phosphorylation of *Mycobacterium tuberculosis* protein tyrosine kinase A PtkA by Ser/Thr protein kinases. Biochem. Biophys. Res. Commun. 467, 421–426. 10.1016/j.bbrc.2015.09.124 26417687

