# Supplementary material for: Eukaryotic-Type Ser/Thr Protein Kinase Mediated Phosphorylation of Mycobacterial Phosphodiesterase Affects its Localization to the Cell Wall
Source: Front Microbiol. 2016 Feb 9;7:123. doi: 10.3389/fmicb.2016.00123 (PMC4746578; doi:10.3389/fmicb.2016.00123)
Supplement: Supplementary file 4 [file DataSheet1.doc]

***Supplementary Materials***

**Eukaryotic-Type Ser/Thr Protein Kinase Mediated Phosphorylation of Mycobacterial Phosphodiesterase Affects its Localization to the Cell Wall**

***Neha Malhotra and Pradip K. Chakraborti^*^***

*CSIR-Institute of Microbial Technology, Chandigarh, India*

****Correspondance:*** Pradip K. Chakraborti, CSIR-Institute of Microbial Technology, Sector 39A, Chandigarh 160 036, India.

*e-mail:* [pradip@imtech.res.in](mailto:pradip@imtech.res.in)

**Supplementary Figures**

**
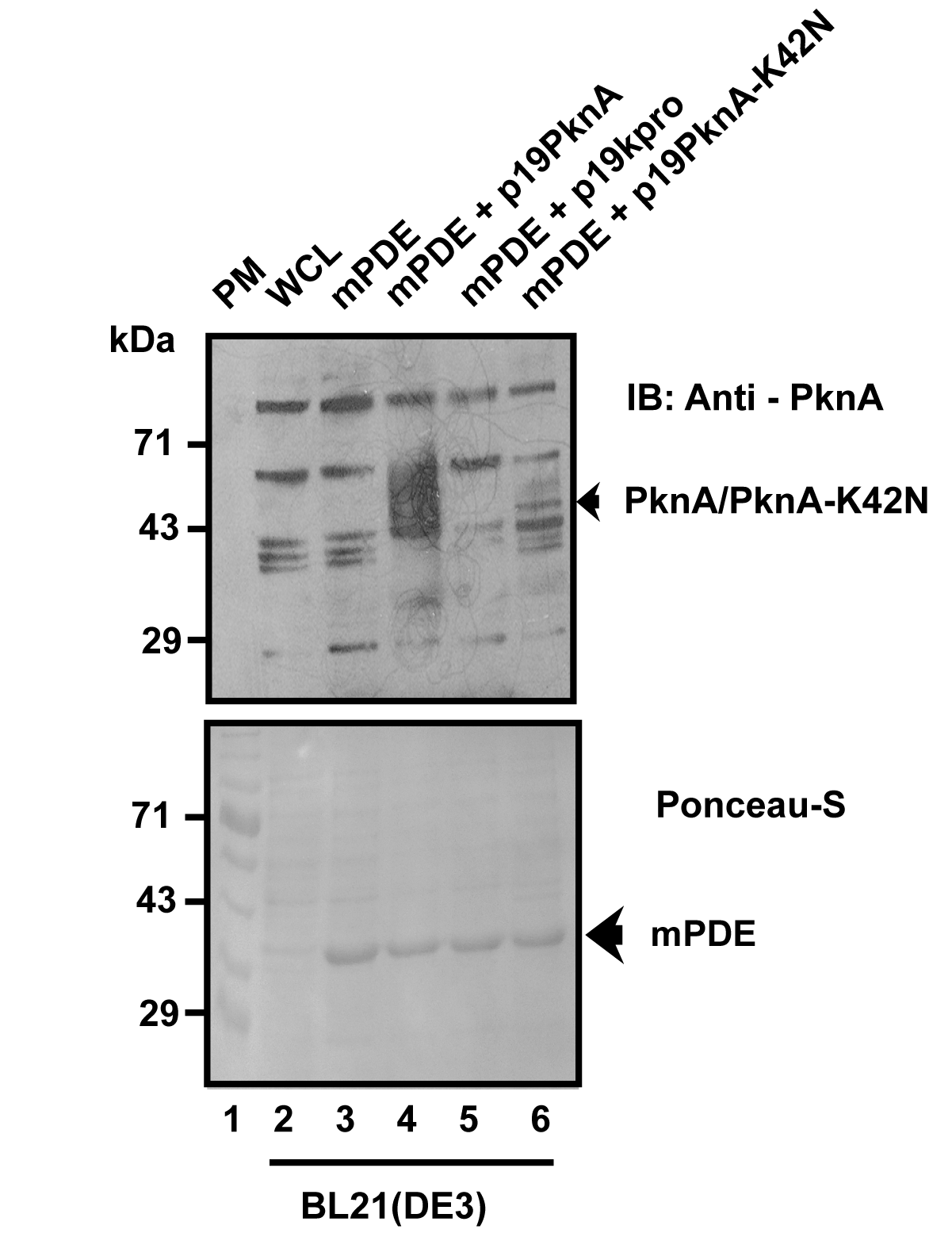
**

**FIGURE S1 | *In vivo* phosphorylation of mPDE.**  BL21(DE3) cell lysates expressing His_6_-mPDE along with either p19kpro-PknA or empty vector (p19kpro) or kinase dead mutant (p19kpro-PknA-K42N) were probed with anti-PknA antibody (upper panel) while ponceau-S stained blot (lower panel) served as loading control. BL21(DE3) whole cell lysate (lane 2) was used as internal control for the experiment. Notations used: WCL, whole cell lysate; PM, pre-stained marker


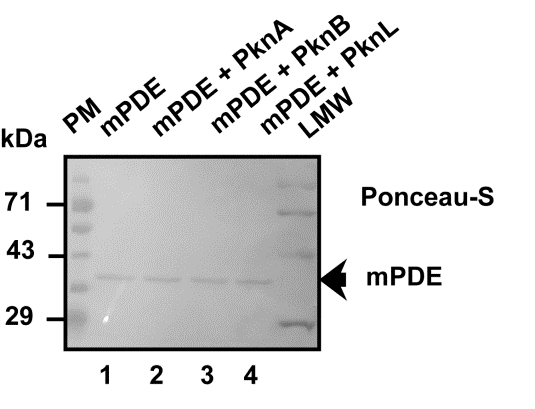


**FIGURE S2 |** **SDS-PAGE of the mPDE stained by Ponceau-S.** Recombinant His-tagged mPDE protein purified using Ni-NTA affinity chromatography of lysates from BL21(DE3) cells expressing either PknA or PknB or PknL in pMAL-c2 vector was resolved in 10% SDS-PAGE gel. Ponceau-S staining of the immunoblot probed with anti-phosphothreonine served as a loading control for **Figure 4B**. Bands represent mPDE proteins in each lane. Numbers indicate molecular mass of pre-stained molecular weight marker in kilo-dalton. Notations used: PM, pre-stained marker; LMW, low molecular mass marker.


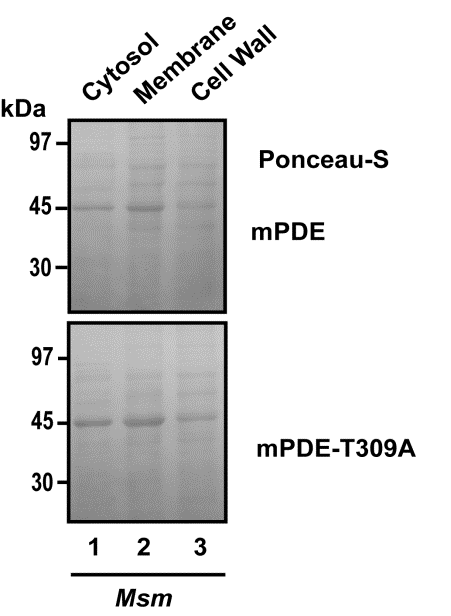


**FIGURE S3 | SDS-PAGE of the mPDE in *M. smegmatis* sub cellular fractions stained by Ponceau-S.** pVV2-mPDE or pVV2-mPDE-T309A were expressed in *M. smegmatis*. Following sub-cellular fractionation, samples (10 µg protein/slot) were resolved in 10% SDS-PAGE. Figure represents Ponceau-S stained blot used for western blotting with anti-His antibody (**Figure 7A**). Upper and lower panels denote sub-cellular fractions of wild-type and mPDE-T309A. Notation used: *Msm*, *M. smegmatis*.

**Supplementary Table Legends**

**Table S1 | Protein-pilot analysis of phosphorylated mPDE.** Phosphorylated mPDE obtained when co-expressed with pMAL-PknA in BL21(DE3) cells (8 µg each) was hydrolysed in ammonium bicarbonate buffer at 37˚C for 16 h by in-solution trypsin digestion method. Tryptic peptides were subjected to LC-MS/MS and analysed using ‘Protein-pilot’ software. LC-MS/MS spectrum representing the b and y ions shown here is an outcome of four independent experiments from two different preparations.

**Table S2 | Identity of ~70 kDa band:** *M. smegmatis* cell wall expressing pVV2-mPDE-T309A (~25 µg) was resolved in SDS-PAGE and visualized by Coomassie staining. Molecular mass region corresponding to the band obtained at ~70 kDa (65-75 kDa) with anti-His antibody in *M. smegmatis* cell wall expressing mPDE-T309A was excised and trypsinized in-gel using protocol mentioned elsewhere. LC-MS/MS of the tryptic digest followed by its analyses using ‘Protein-pilot’ software resulted in the detection of mixture of proteins. **(A)** and (**B**) represents data from two independent experiments.

**Table S3 | Identity of mPDE protein in *M. smegmatis* cell wall by LC-MS/MS.** *M. smegmatis* cell wall expressing pVV2-mPDE (~25 µg) was resolved in SDS-PAGE and visualized by Coomassie staining. Molecular mass region corresponding to mPDE (32-40 kDa) was excised, trypsinized in-gel using protocol mentioned elsewhere and used for LC-MS/MS. Data with a cut-off 0.5 (threshold reduced for obtaining peptides with low intensities as well) was analysed utilizing ‘Protein-pilot’ software. **(A)** Analyses resulted in the identification of mPDE protein in *M. smegmatis* cell wall highlighted in purple at 17^th^ cell. **(B)** Five peptides (highlighted in different shades) for mPDE were obtained following fragmentation and are shown with their respective confidence. **(C)** b and y ions indicated in green for each peptide along with their fragmentation spectra are represented. Peptide sequence ‘IAESGIFIEPSR’ showed 100% identity to the region of mPDE. Reduced peak intensity as seen in fragmentation spectra is very likely because specific band for over-expressed mPDE was not visible with Coomassie staining of *M. smegmatis* cell wall.
